# The Interplay Between Esophageal Adenocarcinoma and Its Tumor Microenvironment: Toward Innovative Therapies

**DOI:** 10.3390/cells14231895

**Published:** 2025-11-28

**Authors:** Rodanthi Fioretzaki, Eleni-Myrto Trifylli, Panagiotis Sarantis, Nikolaos Charalampakis, Konstantinos Christofidis, Markos Despotidis, Michalis V. Karamouzis, Stratigoula Sakellariou, Dimitrios Schizas

**Affiliations:** 1 First Department of Surgery, National and Kapodistrian University of Athens, Laikon General Hospital, 11527 Athens, Greece; rodanthifioretzaki@gmail.com (R.F.); markosd1995@yahoo.gr (M.D.); schizasad@gmail.com (D.S.); 2 Institute of Molecular Medicine and Biomedical Research (IMBE), 11527 Athens, Greece; panayotissarantis@gmail.com (P.S.); mkaramouz@med.uoa.gr (M.V.K.); 3 Department of Biological Chemistry, School of Medicine, National and Kapodistrian University of Athens, 11527 Athens, Greece; 4 Academic Department of Internal Medicine, General and Oncology Hospital “Agioi Anargyroi”, National and Kapodistrian University of Athens, Timiou Stavrou 14, 14564 Kifisia, Greece; 5 Department of Medical Oncology, Metaxa Cancer Hospital of Piraeus, 18537 Piraeus, Greece; nick301178@yahoo.com; 6 First Department of Pathology, School of Medicine, National and Kapodistrian University of Athens, 11527 Athens, Greece; cchristof@med.uoa.gr (K.C.); sakelstrat@med.uoa.gr (S.S.)

**Keywords:** esophageal cancer, esophageal adenocarcinoma, tumor microenvironment, gastrointestinal tumors, immunotherapy

## Abstract

Esophageal cancer (EC) is a highly aggressive gastrointestinal malignancy, with a notable increase in incidence over recent decades, representing a significant global health burden. The main histological subtypes are esophageal adenocarcinoma (EAC) and esophageal squamous cell carcinoma (ESCC), with the former being closely associated with gastroesophageal reflux disease, Barrett’s esophagus, and obesity, and its incidence continues to increase in Western populations. The rising incidence of EC, combined with poor survival rates, underscores the need for new therapeutic approaches. A deeper understanding of the molecular basis of this prevalent malignancy may open new avenues for optimal therapeutic strategies, with immunotherapy now central in several clinical trials. Understanding the interplay between the tumor microenvironment (TME) and disease progression is pivotal for managing this malignancy, which remains highly challenging. This review highlights the role of the TME in EAC progression and drug resistance, and recent therapeutic advances.

## 1. Introduction

Esophageal cancer (EC) is one of the most aggressive gastrointestinal malignancies and shows a steadily rising incidence, particularly in high-income Western countries. According to GLOBOCAN 2022, EC remains a significant public health challenge, with 511,000 new cases reported worldwide [1]. Among its principal histological subtypes, esophageal adenocarcinoma (EAC) represents around 15%, which is strongly associated with gastroesophageal reflux disease (GERD), Barrett’s esophagus (BE), and obesity [2].

Its incidence increases with age, most frequently affecting patients between 60 and 79 years old, and shows a marked male predominance [1,2]. As noted, GERD and BE are the major precursor lesions of EAC. Multiple molecular mechanisms promote inflammation and genomic instability, contributing to the development of metaplasia, progression to dysplasia, and ultimately EAC [3].

The tumor microenvironment (TME) surrounds and supports cancer cells and plays a pivotal role in the progression of EAC. It contributes to invasion, migration, neoangiogenesis, and metastasis, as well as drug resistance. This dynamic and complex entity is composed of (i) extracellular matrix; (ii) immune cells, including: tumor-associated macrophages (TAMs), tumor-infiltrating lymphocytes (TILs), myeloid-derived suppressor cells (MDSCs), T lymphocytes [CD8^+^ cytotoxic T cells, CD4^+^ helper T cells, T regulatory cells (Tregs)], B cells, Natural killer (NK) cells and dendritic cells; (iii) stromal cells including cancer-associated fibroblasts (CAFs); (iv) tumor-secreted molecules, such as growth factor FGF (Fibroblast Growth Factor), VEGF (Vascular Endothelial Growth Factor), and EGF (Epidermal Growth Factor), FGF (Fibroblast Growth Factor), chemokines, pro-inflammatory cytokines (e.g., IL-6, TGF-β, TNF-α), extracellular vesicles, and several enzymes such as matrix metalloproteinases (MMPs); and (v) blood vessels, generated via neoangiogenesis [4]. A better understanding of the role of TME and its components’ implication in EAC progression and drug suboptimal efficacy will open new horizons for an efficacious management of EAC patients. This review will shed light on the pivotal role of the TME in EAC progression and drug resistance, and it will provide a concise overview of novel therapeutic modalities and druggable targets.

## 2. The Role of TME in EAC Pathogenesis

### 2.1. An Overview of Anti-Cancer Immune Response Steps and Tumor Escape

The anti-neoplastic immune response is a multi-step procedure, including (i) elimination, (ii) the equilibrium phase, and, when immunosurveillance fails, (iii) tumor escape. Immune cells recognize the cancer cells through their surface neoantigens, defining the so-called elimination phase. The equilibrium phase occurs when tumor cell growth and immune-mediated elimination are in equilibrium. Nevertheless, when tumor cells escape tumor immunosurveillance, they continue to proliferate, defining the so-called tumor immune escape phenomenon [4,5,6].

### 2.2. An Overview of EAC TME and Its Components

The TME plays a pivotal role in esophageal carcinogenesis, tumor immune escape, disease progression, and drug resistance. This dynamic complex is closely implicated in multiple interactions among the cellular, non-cellular, and soluble biomolecules that comprise it, promoting EAC cell proliferation, invasion, migration, neoangiogenesis, and metastatic dissemination. Patients with EAC often show suboptimal or limited response to anti-neoplastic therapy, with their response being quite heterogeneous, underlining the major influence of TME in treatment response and prognosis [7]. In this paragraph, we present an overview of TME components and their implication in EAC [8,9].

The **extracellular matrix (ECM) in EAC** contains several structural proteins, such as elastin and collagen types I, II, and IV, with type I being the most abundant. Type I collagen, which is a product of CAFs, has a crucial role in bridging gaps between cancer cells and supporting both stromal and cancer cells. E-cadherin is another cell-to-cell-adhesion protein whose downregulation weakens cell adhesion, facilitating cancer cell invasion, migration, and dissemination [10]. Additionally, MMPs such as MMP-2 and -9 are proteolytic enzymes with a crucial role in ECM remodeling and disease progression, promoting cancer cell proliferation, neoangiogenesis, and migration. Meanwhile, it has to be noted that MMPs overexpression is closely related to worrisome disease prognosis [10,11]. Interestingly, adipocytes within the tumor stroma release MMPs (-2, -3, -7, and -9) and lysyl oxidase (LOX), which significantly modify and degrade the ECM, creating migration pathways for cancer cells [10,11]. Furthermore, it has to be underlined that the role of ECM in tumor behavior is crucial, as it not only provides structural support, but also ECM proteins are implicated in cell-adhesion, tumor cell proliferation and survival, invasion, migration, as well as neoangiogenesis via serving as a support system for endothelial cells and metastatic dissemination, as described above [12]. The ECM also plays a key role as a storage site for several growth factors, including FGF, VEGF, TGF-β, and PDGF, which act as tumorigenic molecules and promote desmoplasia. Additionally, these factors influence the anti-neoplastic immune response and neoangiogenesis. Interestingly, when ECM is dense, it prevents the delivery, diffusion, and penetration of therapeutic agents to cancer cells, leading to drug resistance [9,10,11,12,13,14,15]. A denser ECM induces activation of several signaling pathways, including YAP/TAZ and integrin-FAK pathways, which promote proliferation and invasion in EAC. ECM-derived fragments such as matrikines are also implicated in signaling pathways that enhance neoangiogenesis and metastatic dissemination. In addition, cytoskeleton alterations in EAC cells are impacted by a wide variety of proteoglycans, glycoproteins, and other structural proteins such as fibronectin, vitronectin, laminin, integrins, galectin, hyaluronic acid, elastin, as well as dermatan sulfate, which are also found in the ECM of EAC, promoting tumor cell adhesion and migration [8,9]. Other components, such as tenascin-C and osteopontin that facilitate adhesion, migration, metastatic dissemination, and cancer cell survival, respectively [13,14].

**Cellular components in EAC TME:** Several cells within TME are closely implicated in cancer progression, including CAFs, MDSCs, CSCs, Bregs, Tregs, DCs, TILs, as well as TAMs, NK, and ECs. These cells secrete soluble biomolecules, extracellular vesicles (EVs) embedded with a wide variety of pro-tumorigenic cargoes, growth factors, chemokines, and cytokines. These soluble mediators can potentially enhance tumor aggressiveness, promoting cancer cell progression, invasion, migration, and metastasis [16].

- **CAFs in EAC:** They have a key role in secreting several growth factors such as VEGF and TGF-β, which promote the development of an immunosuppressive niche, and enhanced tumor invasiveness (MMP-3–mediated ECM modification via E-cadherin degradation) and drug resistance. However, CAFs can also exhibit a tumor-suppressor role via TGF-β secretion that limits the distant dissemination of tumor cells [17]. Furthermore, CAFs constitute a major factor in drug resistance in EAC TME, while also promoting stromal desmoplasia and neoangiogenesis, and are closely associated with poor survival.
- **MDSCs** have an oncogenic role in TME, as they promote tumor growth and progression by mediating neoangiogenesis (secretion of MMPs, VEGF, and cytokines implicated in angiogenesis) and epithelial–mesenchymal transition (EMT) (secretion of IL-6, TGF-β, and S100A8/A9). In addition, MDSCs suppress anti-neoplastic immune responses of cytotoxic CD8+ T and NK cells, while they promote Tregs, leading to the tumor escape phenomenon. Another effect of MDSCs is the release of cytokines that promote inflammation and tumor progression, such as TNF-α, IL-10, and IL-1β, while they encourage invasion, migration, and metastasis via MMPs. Moreover, they interact with CSCs, mediating their survival, resistance to therapeutic agents, and self-renewal [18]. MDSC-targeted therapeutic strategies, such as Chemokine Receptor (CXCR2) antagonists, are promising strategies, considering that MDSCs significantly promote EMT, tumor neoangiogenesis, and metastatic dissemination [19].
- **B-cells in EAC** are adaptive immune cells, which produce antibodies against the neoantigens of tumor cells, aiming at their elimination. In contrast, Bregs (their subtype) exhibit a tumor-promoting effect, suppressing the innate immune system [20].
- **CD8+ and CD163+ T cells in EAC:** Cytotoxic (CD8+) T-cells are innate immune cells, which recognize tumor cells by their neoantigens through T-cell receptor (TCR) and MHC-I tumor neoantigen interaction. These immune cells exhibit their cytotoxic effect that induces tumor lysis, as well as produce interferon-γ that limits angiogenesis. The increased amount of these cells is closely associated with a more favorable prognosis [21]. EAC’s TME is considered predominantly immunosuppressive. In contrast, some EAC cases present a largely inflamed phenotype, characterized by a high and low number of immune cells, such as CD8 and CD163-positive T cells, respectively, which exhibit anti-tumor effects associated with better outcomes and prognosis [22]. On the other hand, the former subgroup presents a worrisome prognosis, exhibiting an increased number of pro-tumor immune cells, such as Tregs, and a lower number of cytotoxic T cells. Indoleamine-2,3-dioxygenase 1 (IDO) inhibitors are considered promising for targeting T-cell suppression [23].
- **T helper (CD4^+^) cells (Th1/2/17, Tregs) in EAC** are immune cells that release several pro-inflammatory cytokines. These cells have some subtypes that either suppress or promote tumor progression, with Th1 acting as a suppressor and Treg, Th-2, and Th-17 (sometimes) as promoters. Th1 secretes TNF-α and IFN-γ cytokines that enhance and stimulate CD8^+^ cells, and macrophages (anti-tumor phenotype, M1). Meanwhile, Tregs either constitute differentiated T-cells or they originate centrally by the thymus, presenting an increased expression of several molecules such as Cytotoxic T-Lymphocyte (CTLA-4) and Programmed cell death protein 1(PD-1), which are immune checkpoints (ICs), FOXP3, as well as they release increased amounts of cytokines such as IL-2, -10, -35, and TGF-β. Tregs interact with cancer cells, promoting tumor progression, as well as with TME components, including EC for neoangiogenesis, CAFs, and stromal cells. At the same time, they suppress anti-tumor immune responses by inhibiting the function of CD8+ T cells (via FOXP3 overexpression) and NK cells (via IL-2 release). Additionally, Th2 promotes the M2 pro-tumor phenotype of macrophages, which exhibit a tumorigenic action, as well as enhance immunosuppression. Lastly, Th17 either acts as a tumor promoter, facilitating neoangiogenesis, inflammation, and tumor cell progression, or as a suppressor [24,25]. Inflamed EAC phenotypes with abundant CD8^+^ and Th1, releasing anti-tumor cytokines such as IFN-γ and TNF-α, exhibit a more intensive immunosuppression, whereas the other non-inflamed subgroup presents with higher amounts of Tregs, and PD-L1/PD-1 overexpression [26,27].
- **DC:** These are innate immune cells that have a key role in TME, as they constitute APCs, which present the tumor cells (neoantigens) to cytotoxic T cells and stimulate the recruitment of other immune cells via cytokine release and enhance the anti-tumor response via secreting IFN-α/β. DCs usually remain immature within TME, a phenomenon that significantly impairs the anti-neoplastic immune response. Their dysfunction is notably attributed to several biomolecules, which are secreted by cancer cells (e.g., TGF-β), while DCs induce suppression of cytotoxic cells and induction of Tregs [28]. Meanwhile, when immature DCs are notably increased in EAC TME, they are correlated with a worrisome prognosis and TME immunosuppression [29].
- **Neutrophils** are innate immune cells in TME, which present two phenotypes: the (i) N1 (anti-tumor phenotype) that produces reactive oxygen species (ROS), tumor necrosis factor-α(TNF-α), and intercellular adhesion molecule 1 (ICAM-1). N1-phenotype limits immunosuppression and promotes immune cell recruitment for tumor cell destruction. On the other hand, (ii) N2-phenotype (pro-tumor), promotes cancer cell progression, neoangiogenesis (release of VEGF), invasion, migration, and distant dissemination (release of MMP-9), as well as suppression of T cells. However, their tumor-suppressive role is presented only in the early stages of tumor development [30].
- **TAMs** are innate immune cells within TME that have a binary role and phenotype, including the M1 and M2. The former phenotype is anti-tumorigenic, while the latter is pro-tumorigenic. Overexpression of TAMs is relatively associated with poor prognosis, as they release several cytokines and angiogenic mediators that promote neoangiogenesis. Among TAM-secreted molecules, cathepsins and MMPs induce ECM degradation, as well as activation of CAFs, which lead to ECM remodeling. Nevertheless, they are antigen-presenting cells (APCs), which also have anti-tumorigenic effects [31]. TAMs are significantly increased in the EAC-TME, a condition that is closely correlated with worrisome prognosis and TME immunosuppression [29]. TAM modulators such as Colony-stimulating factor-1 receptor (CSF1R) inhibitors are evaluated for the reprogramming or depletion of TAMs [32].
- **NK cells** are innate immune cells that induce tumor lysis directly (perforin) or indirectly, promoting tumor cell destruction and limiting tumor progression. These immune cells recognize cancer cells through their decreased MHC-I expression and induce their apoptosis by releasing perforin or other granzymes. NKs, like cytotoxic T cells, secrete IFN-γ, which enhances immune cell recruitment and anti-neoplastic immune response. However, NK cells can be dysregulated, leading to cancer progression and impaired immunosurveillance. Meanwhile, their action can also be impaired by tumor-secreted biomolecules, such as TGF-β, PD-L1, and IL-10, as well as under hypoxic conditions [33].
- **ECs in EAC** are closely implicated in neoangiogenesis, with VEGF being the key mediator of this pathway. Meanwhile, ECs also have a key role in EMT modulation by releasing cytokines (e.g., TGF-β, IL-6, etc.) and growth factors (e.g., VEGF, PDGF, etc.). These growth factors, such as PDGF, VEGF, and FGF, play a key role in the formation of new vessels that supply tumors. However, these newly developed vessels present an altered wall integrity, a phenomenon that facilitates extravasation, intravasation, migration, and metastatic dissemination of tumor cells. Nevertheless, ECs are not only implicated in neoangiogenesis and EMT modulation, but they are also implicated in tumor escape, as they express PD-L1, which interacts with PD-1 on T-cells, leading to the impairment of immune response. On top of that, activated ECs also express intercellular adhesion molecule-1 (ICAM-1), which is an adhesion molecule that interacts with lymphocyte function–associated antigen-1 (LFA-1) on T cells, typically inducing immune cell recruitment within TME. However, the expression of ICAM can be modified by tumor cells, leading to the impairment of the anti-cancer immune response. In the same manner, EC also expresses another adhesion molecule, such as vascular cell adhesion molecule-1 (VCAM-1), which is bound to monocytes and T-cells. This phenomenon can be exploited by tumor cells for their metastatic dissemination as they express integrins, which can bind to the VCAM-1 that is generally expressed on ECs, promoting cancer cell extravasation and migration to distant sites [34,35].
- **Esophageal CSCs:** These cells originate either from cancer cells that have exhibited plasticity or from physiological stem cells that are converted into CSCs due to epigenetic (e.g., dysfunctional DNA repairing) and genetic alterations (e.g., gene mutations) [36]. CSCs exhibit distinctive characteristics, such as migratory and invasive behavior, increased proliferation due to modified signaling pathways (resulting from genetic aberrations), and increased survival, which is mediated by anti-apoptotic protein overexpression. At the same time, they escape anti-cancer immune responses, and they exhibit resistance to chemotherapy by expressing drug-resistant proteins and efflux pumps. Meanwhile, it has to be underlined that they significantly influence cancer initiation, progression, metastasis, therapeutic resistance, and recurrence in EAC. CSCs may be distinguished and segregated using distinct cell surface and internal markers, including CD44, ALDH, Pygo2, MAML1, Twist1, Musashi1, CD271, and CD90. The isolation of side population (SP), a subpopulation of cells that reject dyes, has been employed to extract CSCs from esophageal cancer. SP cells have stem-like characteristics, including EMT, resulting in increased resistance to radiotherapy compared to parental cells [37]. Esophageal CSCs govern innate and acquired resistance to chemotherapeutic agents, such as 5-FU and cisplatin, in EAC. Recently, a correlation between miRNA expression and chemoresistance in esophageal CSCs has been demonstrated. CSC populations exhibiting heightened tumorigenicity arise when cancers proliferate and encounter therapeutic challenges, such as targeted therapies, cytotoxic drugs, or radiation. Consequently, the elimination of CSCs or the attenuation of their malignant and stem-like characteristics may lead to more effective therapy strategies [38].
- **Soluble mediators:** TME cellular components (tumor, stromal, and immune cells) release several soluble mediators, such as pro-inflammatory cytokines, chemokines, growth factors, extracellular vesicles, large oncosomes (1000–10,000 nm), with the latter being oncogenic molecules secreted by tumor cells that induce TME modification and tumor progression via their cargo delivery to several types of recipient cells. Oncosomes (100–400 nm) are also some tumor-derived EVs that contain cancer-cell-derived bioactive molecules, such as growth factors (e.g., TGF-β1, VEGF, etc.), non-coding RNAs, oncogenes that enhance tumor progression, migration, TME modification, ECM remodeling, modulation of Treg and cytotoxic T-cells function, neoangiogenesis, and metastasis, as well as drug resistance [39]. Among these soluble mediators, growth factors such as VEGFA, TGF-β, and PDGF induce new “leaky” vessel formation, immunosuppression, and ECM remodeling, respectively, as well as EGF and FGF, which enhance cancer cells’ proliferation, survival, and invasion, as well as stroma modification, accordingly. VEGFA is regarded as the most effective pro-angiogenic factor. In both physiological and pathological conditions, VEGF-receptors, particularly VEGFR2, promote endothelial cell (EC) proliferation, migration, and survival [40]. In case of malignancy, it is essential for angiogenesis and tumor proliferation. Meanwhile, it has been demonstrated that it is significantly upregulated in neoplastic tissues and correlated with aggressive characteristics. VEGFA overexpression is also detected in lymph node metastases of EAC, with some studies demonstrating VEGFA mutation being identified in 30–60% of metastasized EAC cases, suggesting its involvement in tumor metastasis. At the same time, it is correlated with worse outcomes, suggesting the potential use of anti-VEGF agents for treating EAC patients who present it, while the expression of VEGF-A and -C is elevated throughout the transition from BE to EAC, indicating their potential association with metastasis and advanced disease stages [41,42].
  Recent investigations have extensively focused on identifying potential molecular markers that predict treatment response and prognosis in EC. Several other angiogenic factors, except the most studied VEGF, are associated with tumor development, growth, and invasion, such as HGF, angiopoietin-2, IL-6, and TGF-β1. Functional genetic variations that influence the expression of angiogenic mediator genes, protein synthesis, and angiogenesis are linked to an elevated risk of malignancies, including EAC [43]. Ardeleanu et al. conducted a study on neoangiogenesis in EAC, involving 40 surgically treated patients. The research identified an axis comprising three components: inflammatory infiltrate, neoangiogenesis, and fibrosis, with notable variations across the three levels of differentiation. An increase in tumor microvascular density was positively correlated with histological grading. Additionally, increased microvascular density proved to be inversely related to cell differentiation and directly related to malignancy risk [44]. Metabolic mediators are also released, such as MMPs, lactate, and indoleamine 2,3-dioxygenase (IDO), with the MMPs promoting ECM degradation, tumor invasion, and metastasis, and the IDO promoting immunosuppression. At the same time, the latter suppresses the function of T cytotoxic cells. Chemokines are closely implicated in cell recruitment, with CCL-2, -5, -9, -10, and -12 being upregulated, while CXCL-9 and -10 being downregulated. The downregulation of CXCL9 and CXCL10 leads to the impairment of cytotoxic NK and T cell recruitment, with tumor cells escaping immunosurveillance. Meanwhile, upregulation of CXCL-2, -5, and -12 leads to the recruitment of immunosuppressive cells such as TAMS, Tregs, and MDSCs. Lastly, Tregs secrete tumor-promoting cytokines such as TGF-β, IL-10, and IL-6, whereas tumor-suppressing CD8+ and NK cells secrete IFN-γ, TNF-α, etc. [45,46,47].
- **Hypoxia in ΕAC’s TME:** An essential aspect of cancer growth is low oxygen tension in the core of solid tumors. Hypoxia is a critical microenvironmental factor that influences tumor development and aggressiveness. The concentration of oxygen regulates a cell’s propensity for specific metabolic pathways, determining its pace of proliferation and invasion. When the tumor reaches a critical size, its core experiences hypoxia. Under these hypoxic conditions, hypoxia-inducible factor-1α (HIF-1α), which is usually degraded rapidly, becomes stabilized. Hypoxic signaling interacts with other pathways, upregulating pro-angiogenic genes, such as cytokines and growth factors, which initiate an angiogenic response. Cellular function is sustained via transcriptional and translational activities, which are energy-demanding processes. Tumor cells redirect their efforts from standard maintenance to activated specialized pathways for energy conservation under hypoxic conditions. Hypoxia-sensing systems trigger biological responses that influence cell survival, resulting in treatment-resistant tumor subpopulations or cell death. The hypoxic conditions within solid tumors constitute a critical microenvironmental factor that affects tumor progression and malignancy. The concentration of oxygen influences a cell’s preference for specific metabolic pathways, thereby affecting its proliferation and invasiveness rates [48,49]. Pseudohypoxia enables cancer cells to initiate hypoxia-dependent pathways despite oxygen abundance. In addition, Tan et al. examined the identification of prognostic hypoxia-related genes in EC. The hypoxia model for EAC included three genes: PGK1, PGM1, and SLC2A3. This research demonstrated that individuals of the high-risk cohort exhibited reduced survival rates and significant infiltration of immune cells. Data also indicated a correlation between oxygen deprivation, prognosis, and immune cell infiltration in patients with EAC [7,50]. In Table 1, we present the TME components and their implication in TME.

## 3. TME Modulation as a Therapeutic Strategy in EAC—Clinical Trials

Some of the therapeutic strategies that will be discussed in this paragraph are: immune checkpoint inhibitors (ICIs) [anti-Programmed Cell Death Protein-1 (PD-1)/anti-Programmed Death-Ligand 1 (PD-L1), anti-Cytotoxic T-Lymphocyte Antigen-4 (CTLA-4)] with or without the combination of radiotherapy/chemoradiotherapy and conventional chemotherapy, as well as other approaches that target CAF-related ECM remodeling, immunosuppressive cells, and angiogenesis [51]. Some of the druggable targets are the following:

- **CD136-positive T cells:** The decreased number of CD136-positive T cells has been evaluated as a druggable target in the phase 2 AGITG DOCTOR trial (ACTRN12609000665235), based on the data acquired from the Australian New Zealand Clinical Trials Registry. Patients in this trial presented resectable EAC, and chemoresistance to cisplatin and 5-FU. They were randomized into two arms: one received docetaxel and the aforementioned chemotherapeutic strategy, and the other received this treatment regimen (2 cycles) combined with PET-guided radiotherapy (45 Gy total dose). The latter group of patients presented a higher 5-year overall survival (OS) and histopathological response in comparison to non-responders to chemotherapy; however, with higher grades of toxicity (grade 3/4) [52].
- **PD-L1 expression:** An ongoing study is STAR-221 (phase 3, NCT05568095), which evaluates the combination of domvanalimab (anti-TIGIT), zimberelimab (anti-PD-1), and chemo versus nivolumab (anti-PD-1) and first-line chemotherapy [FOLFOX or CAPOX (capecitabine, Oxaliplatin)] for advanced, metastatic, and unresectable GEJ or EAC patients; however, the primary results have not yet been reported [53]. Nevertheless, the EDGE-Gastric study (phase 2, NCT05329766) demonstrated a higher overall response rate (ORR) for EAC, GEJ, and GC patients who received the combination of domvanalimab, zimberelimab, and first-line chemotherapy [54]. In addition, the MATTERHORN Trial (phase 3 trial, NCT04592913), in which the combination of durvalumab (anti-PD-L1) plus FLOT (5-FU, leucovorin, oxaliplatin, docetaxel) is used as perioperative (pre- or postoperative) treatment for patients with resectable GEJ/GC, demonstrated improvement of OS and increased pathologic complete response [55,56]. The combination of durvalumab with FLOT achieved a statistically significant OS improvement based on the final OS analysis that has been reported at the latest ESMO 2025. DANTE (IKF-s633) trial (phase 2/3, NCT03421288) evaluated the addition of atezolizumab (anti-PD-L1) plus FLOT, in comparison to FLOT, for the same group of patients, who significantly benefited from the addition of atezolizumab (especially for those who had overexpression of PD-L1 and high MSI status) [57]. MORPHEUS-EC (Phase 1b/2, NCT03281369) evaluates the combination of atezolizumab, tiragolumab (anti-TIGIT), and chemotherapy versus chemotherapy alone, versus atezolizumab and chemotherapy, with the former combination enhancing survival and tumor response in patients with advanced EC [58]. Meanwhile, SKYSCRAPER-08 (YO42138) trial (phase 3, NCT04540211) has evaluated the combination of atezolizumab, tiragolumab, paclitaxel, and cisplatin, which increased the OS; however, in ESCC patients with unresectable/locally advanced or metastatic disease [59]. PERFECT trial (phase 2, NCT03044613) evaluated five cycles of atezolizumab plus neoadjuvant chemoradiotherapy (paclitaxel, carboplatin, and radiotherapy) with no significant differences regarding patient response and survival rates. Interestingly, patients with higher IFNγ baseline levels showed a greater response, whereas non-responders exhibited overexpression of immune checkpoints and either higher or decreased cytotoxic T cell levels [60].
- **PD-1 expression:** KEYNOTE-975 (phase 3, ongoing, NCT04210115) [45] and KEYNOTE-590 (phase 3, NCT03189719) [61] evaluate another type of ICI, such as PD-1, with the latter trial including patients with EAC and the former advanced EAC and ESCC, supporting first-line pembrolizumab for advanced EC based on long-term analysis [61]. More particularly, KEYNOTE-590 demonstrated a beneficial effect (progression-free and overall survival) for patients who received a combination of pembrolizumab plus chemotherapy [61]. One arm in KEYNOTE-975 receives pembrolizumab and chemoradiotherapy (fluorouracil or derivatives or paclitaxel), whereas the other placebo and chemoradiotherapy, with no primary results being reported [62]. Meanwhile, the final analysis of the phase 3, KEYNOTE-585 (NCT03221426) trial for patients with resectable, locally advanced GEJ and GC did not meet the primary end points, such as the improvement of OS for the experimental Arm (A), who received pembrolizumab, combined with pre-operative, post-operative or maintanance chemotherapy with pembrolizumab versus the control arm, in which chemotherapy was administrated [63]. Another trial (phase 3, CheckMate-649) evaluated Nivolumab (PD-1 inhibitor) in combination with chemotherapy versus chemotherapy alone for patients with GEJ, EAC, gastric cancer (HER2 negative), supporting an improved OS [64]. Meanwhile phase 2, NCT03044613 also evaluated the utilization of neoadjuvant nivolumab (arm A) ± relatlimab (anti-LAG-3) (ARM B) before chemoradiotherapy in resectable stage 2/3 EC or GEJ cancers, before chemoradiotherapy in resectable stage 2/3 esophageal or gastroesophageal junction (E/GEJ) cancers, with the latter regime presenting toxicity, and the former meeting primary safety points, while it has been demonstrated that patients with higher levels of LAG-3 and PD-L1 had a greater response [65].
- **EAC CSCs:** Elimination of CSCs or the attenuation of their malignant and stem-like characteristics may lead to more effective therapy strategies [38], as they influence cancer initiation, proliferation, metastasis, therapeutic resistance (5-FU and cisplatin), and recurrence.
- **VEGF:** Anti-VEGF agents (e.g., ramucirumab, bevacizumab) induce limitation and normalization of tumor vascularity, as well as hypoxia limitation. Some of the already completed trials studying anti-VEGF agents (bevacizumab, ziv-aflibercept) in EAC include: the combination of bevacizumab, cisplatin, irinotecan with pre-operative chemoradiation (phase 2, NCT00570531), which was early terminated as it did not reach the required number of subjects within the timeline [66]. Another trial is the ZAMEGA (NCT01747551), which included patients with refractory GC/GEJ adenocarcinoma. A combination of Ziv-Aflibercept with modified FOLFOX6 (mFOLFOX6, oxaliplatin, leucovorin, and bolus plus infusion 5-FU) versus mFOLFOX6 was evaluated; however, without significant benefits, as it did not improve the efficacy of mFOLFOX6 [67]. The combination of bevacizumab and mFOLFOX6 as a first-line regimen for patients with untreated metastatic GEJ/GC has proven beneficial (higher OS and PFS), and well-tolerated [68]. In addition, the combination of ramucirumab (anti-VEGF2) plus mFOLFOX6 has been evaluated in a phase 2 NCT01246960 trial for advanced EAC/GEJ/GC, compared to mFOLFOX6 and placebo, and it has also demonstrated a higher OS based on exposure-response analysis [69]. Additionally, the AVAGAST trial (phase 3) proved that the addition of bevacizumab to chemotherapy (cisplatin, capecitabine) improved the OS of patients with non-resectable, locally advanced, or metastatic GEJ/GC, with plasma VEGF-A and tumor neuropilin-1 being considered as predictive biomarkers for treatment response in patients being treated with bevacizumab [70]. Meanwhile, the RAINBOW (phase 3, NCT01170663) [71] and REGARD (phase 3, NCT00917384) [72] trials evaluated ramucirumab as a combination with paclitaxel versus paclitaxel and placebo, and ramucirumab monotherapy versus placebo, respectively. RAINBOW showed an improved OS with the addition of ramucirumab to paclitaxel as a second-line strategy [71]. At the same time, REGARD also demonstrated that ramucirumab monotherapy improved the survival of non-naive patients with advanced GC OR GEJ [72].
- **VEGF & anti-PD-1:** The combination of ramucirumab and pembrolizumab (anti-PD-1) has been evaluated in phase 1a/1b JVDF trial for patients with GEJ as second-line (cohort A) (NCT02443324). This combination was considered superior, particularly for patients with tumors expressing PD-L1 [73]. Another phase 2 trial (NCT04632459) evaluates the combination of pembrolizumab with ramucirumab for metastatic GC/GEJ, with no published results [74]. Meanwhile, some of the ongoing trials for EC that are still recruiting are phase 2 NCT06415851 [75] and NCT05163483 [76], with the former evaluating the combination of bevacizumab, chemotherapy, and anti-PD-1 in patients with advanced EC [75], while the latter evaluates the combination of bevacizumab, anti-PD-1, and tucidinostat (HDAC inhibitor) for patients with advanced MSS/MMR EC [76].
- **CAF-targeted strategies**: Phosphodiesterase type 5 inhibitors (PDE5i) have been evaluated, aiming at the enhancement of SOC chemotherapy efficacy in EAC patients [77]. It has been demonstrated that PDE5i limits fibroblast trans-differentiation into CAFs, while, when combined with chemotherapy, it can overcome CAFs-related chemotherapy resistance in EAC [77].
- **IL-6 signaling pathway inhibition** (e.g., via tocilizumab) constitutes another modality, which limits EAC cells’ growth. It minimizes TME immunosuppression and induces resensitization of EAC cells to chemoradiation or chemotherapy in preclinical studies [78].
- **NOX4 blockade** constitutes another approach for CAF-rich tumors, which could improve disease outcomes and minimize CAF-related immunoresistance [79]. However, more preclinical and clinical studies are needed to highlight the promising role of CAF-targeted agents in EAC.
- **CXCR2 antagonists** target the immunosuppressive MDSCs, and they are considered promising for TME modulation in EAC [19].
- **CSF1R inhibitors** are evaluated for the TAMs modulation [32].
- **IDO** inhibitors are promising modalities for targeting T-cell suppression, taking into consideration that IDO1 enzyme has a key TME immunomodulatory role that promotes tumor immune evasion [80].

In Table 2, we demonstrate TME modulation in EAC and therapeutic strategies, including completed and ongoing clinical trials.

## 4. Modulation of the Molecular Background for EAC Therapeutic Management

### 4.1. Genetic Aberrations in EAC Leading to TME Modification

Several genetic aberrations have been identified in EAC, including mutations, amplifications, and deletions, with some having significant relevance to disease prognosis. Notably, loss-of-function mutations in *CDKN2A* and *TP53* are closely associated with a worrisome prognosis and outcomes. Some of the genetic alterations that have been identified in EAC include [81].

#### 4.1.1. *TP53* Mutation (chromosome 17p13.1)

- **Frequency:** Recent genomic studies indicate that TP53 gene mutations are the most common genetic alteration in EAC and BE with dysplasia, with wild-type *TP53* detected in only 30% of EAC [82,83,84]. Mutations in the *TP53* gene commonly occur during BE progression toward carcinogenesis, increasing the risk of advancement by 13.8 times, as reported by Stachler et al. (2015) [85]. Loss of heterozygosity (LOH) of the *TP53* gene and mutations are prevalent in most cases of EAC, accounting for approximately 70% of cases, a phenomenon closely associated with disease progression [85]. A meta-analysis study suggested that these mutations have a significant impact on prognosis and OS in EAC patients, regardless of tumor stage, due to their crucial role in EAC pathogenesis [86,87].
- **Physiological role:** The *TP53* constitutes a tumor suppressor gene, which has a key role in genomic stability, cell cycle regulation, prevention of damaged DNA replication, and multiplication of damaged/aged cells, for which apoptosis is triggered. Meanwhile, it also regulates several metabolic pathways, which are implicated in oxidative stress management [88].
- **Loss of function in EAC:** Genetic instability occurs, and replication of damaged DNA is not prevented [82]. Immunohistopathological observations have shown that p53 staining is mainly enhanced in EAC and dysplastic BE tissues, although some non-dysplastic BE tissues also express it, a phenomenon linked to high risk of progression [87].
- **Role as biomarker:** *TP53* mutation and altered p53 expression could be utilized as a potential marker of early neoplastic development in patients with BE [81,82,83,84,85,86,87,88].
- **Implication in EAC-TME:** It induces extensive modifications in TME in several aspects, including: (i) enhancement of infiltration with immunosuppressive cells (M2 macrophages, MDSCs, and Tregs), via over-secretion of cytokines IL-6, IL-8, TGF-β, and chemokines such as CXCL9, -10, and -12; (ii) suppression of infiltration with anti-tumor cells such as T cytotoxic and NK cells; (iii) failure of VEGF suppression (pro-angiogenic factor), resulting in neoangiogenesis; (iv) promotes hypoxia via enhancement of HIF-1α stability (increased ROS) and subsequently increases VEGFA overexpression and neoangiogenesis; (v) enhancement of IC expression like PD-L1, promoting tumor escape phenomenon and protection of EAC cells from the cytotoxic effects of T cells; (vi) increased fibroblast transformation into CAFs, leading to a denser stroma; (vii) increased excretion of EV- tumor promoting miRNAs (viii) ECM modifications, via MMPs overexpression, leading to EAC cell invasiveness and metastatic behavior; (ix) increased IDO, suppressing of T-cell anti-tumor action [86,89,90,91,92,93].
- **Druggable target:** Several drugs have been identified for their ability to reactivate mutant *TP53*, such as PRIMA-1 and its analogue APR-246 in EAC cells [94,95]. The agents mentioned above constitute the most extensively studied agents targeting *TP53*, with the latter inducing the upregulation of the *TP53* gene, cell-cycle arrest, apoptosis, as well as the enhancement of the inhibitory effects of the chemotherapeutic agents such as cisplatin and 5-FU (synergistic effect) via p53 accumulation in EAC cells. Interestingly, cancer cell sensitivity to APR-246 has been associated with the intracellular levels of mutant p53, whereas knockdown of the TP53 gene has been related to reduced APR-246 activity. Meanwhile, it was also shown that APR-246 not only enhanced the action of chemotherapeutic agents such as cisplatin and 5FU, but also enhanced the anti-tumor response [94]. Nevertheless, APR-246 has shown little cytotoxicity towards normal cells. Phase 1b/2 trial currently assesses its effectiveness in platinum-resistant advanced and metastatic esophageal or GEJ tumors (NCT02999893), in which APR-246 is combined with cisplatin + 5-FU [96]. Meanwhile, another Phase 1b/2 clinical trial (NCT04383938) is underway, combining APR-246 with pembrolizumab in solid tumors. This trial has demonstrated that this combination is well-tolerated and clinically beneficial for these patients [94,95].

#### 4.1.2. Human Epidermal Growth Factor Receptor 2 (*HER2*) or *ERBB2* Gene (chrom. 17q12)

- **Frequency:** *HER2* amplification is identified in ~15–25% of EAC, which is closely related to a poor prognosis, reduced OS, and increased aggressiveness in EAC [97,98]. Meanwhile, HER2 overexpression is relatively rare in non-dysplastic; however, its presence is linked with a higher risk of progression from dysplastic BE to EAC.
- **Physiological role:** It encodes a receptor tyrosine kinase (RTK), which is a member of the EGFR/ERBB family that has a key role in cell growth, differentiation, and survival in signaling downstream pathways of MAPK and PI3K/AKT, MAPK [98].
- **Implication in EAC-TME:** Mutant HER2 induces several TME modifications, including: (i) hypoxia via HIF-1α stability, leading to increased VEGFA and angiogenesis; (ii) induction of endothelial proliferation, overexpression of angiopoietins promoting neoangiogenesis and EMT; (iii) increased release of tumorigenic cytokines like IL-8, IL-6 and chemokines (CCL-2,-5 and CXCL12); (iv) enhanced immunosuppression and tumor escape phenomenon via increasing the recruitment and infiltration of immunosuppressive cells (M2 macrophages, MDSCs, and Tregs) and reducing T-cytotoxic and NK cells; (v) ECM remodeling leading to a denser stroma (increased effect of CAFs, as well as to a degraded ECM via MMPs upregulation), with the former phenomenon leading to drug resistance; (vi) acidization of TME via enhancement of aerobic glycolysis, which is suppresses the action of CD8+ cells; (vii) supply of nutrients for EAC cells; (viii) secretion of EVs (oncosomes) with tumorigenic miRNAs, mRNAs or ICs [99,100,101,102,103,104,105,106,107].
- **Druggable target:** the potential use of HER2 as a druggable target for EAC, and its evaluation is crucial, as anti-HER2 agents (e.g., trastuzumab) could significantly benefit EAC patients [108]. DESTINY-Gastric01 (NCT03329690, phase 2) evaluates trastuzumab deruxtecan (DS-8201a) in patients with HER2-positive tumors (non-resectable or metastatic GEJ adenocarcinoma GC versus physician’s choice (paclitaxel or irinotecan monotherapy or SOC), with the experimental arm having a higher ORR [109]). Meanwhile, DESTINY-Gastric05 (NCT06731478, phase 3) evaluates the safety and efficacy of triplet combination regimen including DS-8201a (ENHERTU, T-DXd), pembrolizumab, and fluoropyrimidine (5-FU or capecitabine) versus first line SOC treatment (cisplatin plus 5-FU or oxaliplatin plus capecitabine), pembrolizumab and trastuzumab for PD-L1 CPS ≥ 1, HER2 positive metastatic or locally advanced, non-resectable GEJ/GC [110]. An already completed trial that tested pertuzumab plus trastuzumab and chemotherapy for previously untreated HER2-positive metastatic GEJ/GC (JACOB trial, phase 3, NCT01358877) did not meet its primary endpoint of OS improvement [111].

#### 4.1.3. *VEGF* Gene (VEGFA; chrom. 6p21.1)

*VEGF* Gene (VEGFA; chrom. 6p21.1) was previously mentioned in Section 2.

#### 4.1.4. *CDKN2A* (Cyclin-Dependent Kinase Inhibitor 2A) Gene (chrom. 9p21.)

- **Frequency:** It is commonly found altered in ~20–30% of EACs, in which *CDKN2A* deletion or mutation is observed [112].
- **Physiological role:** It constitutes another pivotal tumor suppressor.
- This gene encodes p14ARF and p16INK4a proteins, with the former regulating TP53 and the latter inducing the arrest of the cell cycle in phase G1/S. Cell cycle regulation is crucial for cellular proliferation, with cyclins and cyclin-dependent kinases (CDKs) serving critical functions. At the same time, the CDK4–CDK4-CDK4-Cyclin D complex plays a pivotal role in regulating retinoblastoma protein (Rb), which involves phosphorylation and functional inactivation of Rb, ultimately leading to cell cycle S-phase entry [113,114].
- **Loss of function in EAC:** The loss of its function is even more frequently identified in EAC cases, as well as the hypermethylation of its promoter in ~30–60% of EAC and ~15–50% in BE [112]. Paradoxically, early loss of CDKN2A acts as a tumor inhibitor, as it is primarily identified in BE cases with no progression compared to BE with progression to EAC. However, the loss of *CDKN2A* late in the disease is closely associated with a worrisome prognosis based on the findings in the study of Ganguli et al. [112]. EAC overexpresses several cyclins, with cyclin D1 elevated in 25–38% of BE patients and 36–44% in EAC patients. Cyclin E expression is elevated in particular dysplastic BE and 19.0% of EAC cases, often linked to amplification of 19q12, the locus of the cyclin E gene [115]. P27 expression is reduced in most EAC, and in a surgically produced reflux model, p27 knockout mice have higher incidences of BE and EAC compared to wild-type animals [116]. Moreover, cyclin D overexpression is mainly identified in EAC (20–30%) and high-grade dysplasia, and to a lesser extent in non-dysplastic BE. At the same time, EAC influences the tumor suppressors p16INK4a and p14ARF, which are encoded by the CDKN2A gene. The former promotes excessive cellular proliferation by inhibiting cyclin D/CDK4/6 complexes [113,114,115,116,117]. Meanwhile, downregulation of the p14ARF is observed in 20% of BE cases and 75% of EAC cases, disrupting the p53 response [114]. p27KIP1, a cyclin-dependent kinase inhibitor, modulates the cell cycle by inhibiting cyclin E/CDK2 and cyclin D/CDK4 complexes. However, altered gene function leads to several other mutations, such as *TP53* and loss of cell cycle control and CDK4/6 cell-cycle kinases overexpression, which are usually restrained and regulated by CDKN2A, implying the possible utilization of CDK4/6 inhibitors such as palbociclib, abemaciclib, and ribociclib, which could lead to cell cycle arrest in G1 phase [118].
- **Role as biomarker:** Lastly, the absence of p16INK4a protein expression in immunohistochemistry could serve as a potential biomarker for dysplasia and disease progression from BE to EAC [119].
- **Implication in EAC-TME: ***CDKN2A* mutation leads to several TME modifications, including: (i) enhanced immunosuppressive cells (M2 macrophages, MDSCs, and Tregs) infiltration and suppression of cytotoxic NK and CD8+ T-cells; (ii) ECM remodeling with activated CAFs (fibrosis), release of growth factors promoting EMT and angiogenesis, and ECM degradation via MMPs; (iii) hypoxia-induced neoangiogenesis via HIF-1a stability [112,120].
- **Druggable target:** Preclinical studies showed a potential beneficial effect in EAC models in case of the second; however, there was a non-promising impact in case of the former, based on the results of the phase 2 trial, in which palbociclib was used as monotherapy (NCT01037790) [121]. Meanwhile, another phase 1/2 study (NCT03065062) currently evaluates the combination of palbociclib with other agents such as gedatolisib, with the latter being a PI3K/mTOR inhibitor [122]. Another approach involves reactivating CDKN2A through epigenetic modulation before neoadjuvant chemotherapy (NCT01386346, phase 1). This approach has also been evaluated using azacitidine, with tumor hypomethylation serving as an indicator of histopathologic response [123].

#### 4.1.5. *KRAS* Gene (chrom. 12p12.1)

- **Frequency:** It is a crucial proto-oncogene in human malignancies. In contrast, it regulates cell proliferation, differentiation, and survival. KRAS mutations are present in 30% of all [124,125,126] malignancies, with KRAS amplification being prevalent in approximately 17% of EAC cases [127]. KRAS amplification is correlated with lymph node involvement and reduced OS, and a poor prognosis in EAC [126].
- **Physiological role:** It encodes KRAS, a small GTPase that modulates signal transduction pathways (RAS-RAF-MEK-ERK signaling pathway), acting as a “switch” [126].
- **Pathological function in EAC:** It promotes the development of EAC from dysplastic BE.
- **Implication in EAC-TME:** It induces EAC-TME reprogramming, including: (i) recruitment and infiltration of immunosuppressive cells; (ii) suppression of cytotoxic NK and CD8+ T cells, leading to failure of tumor immunosurveillance; (iii) CAFs activation leading to stromal changes and growth factors release (PDGF, TGF-β, etc.) [42,128,129,130].
- **Druggable target:** Some of the ongoing clinical trials examine LY4066434, a Pan-KRAS inhibitor (phase 1, NCT06607185) for advanced solid tumors [131], as well as BI 3706674 (phase 1, NCT06056024) in patients with gastric or esophageal cancer that present KRAS amplification [132].

#### 4.1.6. *SMAD4* Gene (Mothers Against Decapentaplegic Homolog 4, chrom. 18q21.2)

- **Frequency:** Mutations/deletions/LOH are also identified in EAC cases (5–10%) [133,134,135].
- **Physiological role:** The SMAD4 gene encodes the SMAD4 protein, which has a key role as a tumor suppressor, being closely implicated in the transforming growth factor beta (TGF-β)/SMAD signaling pathway, regulating ECM production, cell growth, differentiation, and apoptosis [133,134,135].
- **Loss of function in EAC:** Aberrations in SMAD4 protein function and expression are closely related to poor prognosis, advanced EAC, and metastatic dissemination; however, they are not common in early-stage BE [136]. SMAD4 expression is altered with the loss of its tumor suppressive effect, TGFβ pathway is dysfunctional (TGF-β → SMAD2/3 → SMAD4 altered) in EAC, compared to the normal esophagus and BE, with the function of SMADs often being lost absent in EACs (SMAD2, -4 are mostly affected), a phenomenon that can promote EMT, cancer cell invasion, and metastasis in BE [134,135,136].
- **Implication in EAC-TME:** (i) dysfunctional TGF-β signaling leading to EMT; (ii) recruitment and infiltration of immunosuppressive cells (Tregs, TAMs (M2 macrophages), and MDSCs), which secrete pro-tumorigenic cytokines (IL-6, IL-8, TNF-a) and chemokines (CXCL1), respectively; (iii) suppression of NK and CD8+ T cells; (iv) increased MMPs, leading to ECM degradation; (v) dense, fibrotic stroma via activated CAFs, which release of fibrogenic proteins; (vi) induction of hypoxia, and subsequently neoangiogenesis; (vii) increased EMT; (viii) increased release of EVs that contain pro-tumorigenic and ECM remodeling-related molecules [136,137,138].
- **Druggable target:** Selective targeting of BMP2/4 by specific llama-derived antibodies (VHHs) and C4C4 against BMP4 and C8C8 against BMP2/4 in SMAD4-negative EAC has been evaluated in preclinical studies, such as that of Li, S. et al. [136,139]. More particularly, BMP signaling (non-canonical) is triggered by the overexpressed BMP2 and 4, promoting EAC (SMAD4-negative) aggressiveness; however, the inhibition of BMP2/4 via the VHHs limits tumor growth and increases survival [139]. In addition, it has been demonstrated that EMT in BE is closely related to BMP4 signaling, which leads to disease progression and EAC development [140]. Another preclinical study (organoids) also evaluates the suppression of BMP2/4 in BE, which leads to the epithelial regeneration (squamous) and the limitation of columnar (pathological) epithelium [139].

#### 4.1.7. *ARID1A* (AT-Rich Interactive Domain-Containing Protein 1A) Gene, Located on chrom. 1p36.11

- **Frequency:** It is also found to be mutant in EAC cases (9–15%) [141,142].
- **Physiological role:** *ARID1A* has a key role as a tumor suppressor, as well as a regulator of the DNA repair mechanism, gene expression, and its protein is a member of the SWI/SNF chromatin remodeling complex [141,142].
- **Loss of function in EAC:** Loss of *ARID1A* expression is observed in ~10% of EAC cases, 4.9% in dysplastic BE, and 16% in high-grade dysplasia. In contrast, non-dysplastic tissue shows absent expression based on immunohistochemistry findings on biopsied samples [141,142]. *ARID1A* mutations are identified in the early stage of EAC, with ARID1A deficiency being widespread in disease progression from metaplasia towards EAC development [143]. Meanwhile, the loss of ARID1A expression is related to microsatellite-stable (MSS) in 12% of EAC cases and may co-occur with TP53 mutation. As observed in EAC line cells, the deletion of the *ARID1A* gene results in tumor cell proliferation, growth, and invasion. Consequently, these patients present with a poor prognosis and reduced OS [144].
- **Implication in EAC-TME:** (i) higher amounts of T cell levels are implicated in the tumor escape phenomenon; (ii) ARID1A deficiency is also implicated in fatty acid lipid metabolism, which can also be modulated for EAC treatment. More particularly, it is demonstrated by Zhang, L. et al. that *ARID1A* has a key role in the regulation of the genes implicated in lipid metabolism in EAC, with its knockdown downregulating the genes such as SREBF 1/2, MVD, FASN, and SCD1, which take part in de novo lipogenesis. Interestingly, overexpression of FASN has been demonstrated in both BE and EAC. In contrast, its suppression leads to the suppression of EAC cell proliferation [143]. At the same time, statins have been correlated with a lower risk of EC in BE patients based on meta-analytic data [143,144,145].
- **Druggable target:** ICIs constitute promising therapeutic modalities, with the treatment response being closely related to the levels of T-cells, microsatellite instability, as well as LAG-3 and PD-L1 expression. Based on the findings mentioned earlier, a new study will evaluate the combination of oxaliplatin, simvastatin (HMG-CoA reductase inhibitor), and nivolumab (anti-PD-1) in patients with advanced GC/EC and ARID1A mutation; however, it is not currently recruiting participants [146]. Other trials for ARID1A-mutated tumors are: the ongoing phase 2 NCT05023655, which evaluates the utilization of tazemetostat in solid tumors with ARID1A mutation [147]. At the same time, there is another ongoing trial, which evaluates the single dose dual ICIs (tremelimumab and durvalumab), aiming at the increase of intra-tumoral T cells in ARID1A-mutant GC/EAC/GEJ adenocarcinomas, with the intra-tumoral effector T-cells (CD3/CD8) being evaluated after the single dose (2–6 weeks) versus baseline [148].

#### 4.1.8. *PIK3CA* Gene

- **Frequency:** This mutation is identified in approximately 5–15% of EAC cases, while amplification of the genes that are implicated in the PI3K/AKT/mTOR signaling pathway is also found [149].
- **Physiological role:** It encodes the p110α catalytic subunit of class I phosphatidylinositol-3-kinase (PI3K) and is implicated in PI3K/AKT/mTOR signaling pathway, with its protein being activated when molecules such as cytokines and growth factors bind to RTKs. Typically, it has a crucial role in the control and regulation of cell growth and proliferation, as well as in hypoxia-induced angiogenesis and cell survival [150].
- **Altered function & implication in EAC-TME:** Its mutation could lead to a constant activation of the aforementioned signaling pathway, leading to uncontrolled cell growth, proliferation, angiogenesis, and carcinogenesis. At the same time, it has to be underlined that the PIK3CA mutation has been correlated with a more favorable prognosis in EAC cases (early-stage), compared to other mutations [149], while it is closely related to drug resistance to chemotherapy, as well as other anti-neoplastic agents such as HER-2 inhibitors [149,150].
- **Druggable target:** These mutations have been utilized in several clinical trials as druggable targets. More particularly, PI3KCA inhibitors have already been evaluated for EAC with this mutation. Phase 2 study of BKM120 (NCT01501604) with advanced solid tumors, included EC patients with PIK3CA mutations/amplifications; however, it was withdrawn [151]. Selective PIK3CA inhibitor Inavolisib (GDC-0077) was evaluated in the phase 2 CRAFT trial (NCT04551521), with favorable results in disease management [152]. Meanwhile, the evaluation of PF-07248144 (a selective PI3Kα inhibitor) in the NCT04606446 trial for metastatic solid tumors, including EAC, is still ongoing [153]. In addition, another Pan-PI3K inhibitor, such as Buparlisib (BKM120), has also been examined, although in ESCC cases [154]. Another phase study evaluated CDK4/6 inhibition with palbociclib (NCT01037790) [155,156].

#### 4.1.9. *MYC* Gene (chrom. 8q24)

- **Frequency:** *MYC* amplification is found in a large portion of EAC cases (45.3%) [157,158].
- **Physiological role:** It encodes c-Myc protein, which has a key role as a transcription factor. Typically, it regulates gene expression, cell metabolism, as well as cell apoptosis, growth, and proliferation (cell-cycle regulation G1/S) [157,158].
- **Aberrant function in EAC:** C-myc overexpression is closely related to poor prognosis and uncontrolled cancer cell growth, proliferation, drug resistance, and prolonged survival. It is implicated in the activation (upstream) of several signaling pathways, such as PI3K/AKT, Wnt, and KRAS. It has to be noted that BE neoplastic transformation often leads to abnormal activation of the Wnt/β-catenin signaling pathway, which is crucial for tumor development [157,158]. Nuclear expression of β-catenin is observed in 44–53% of low-grade dysplasia, 42–93% of high-grade dysplasia, and 61–63% of EAC [158]. Nuclear accumulation can occur as a result of APC LOH, a common late event in EAC, either by increasing Wnt ligand expression or by epigenetically silencing Wnt inhibitory factor (WIF1) [159,160,161]. The interaction between β-catenin and E-cadherin enhances signaling. BE and EAC often have decreased membranous E-cadherin, potentially attributable to promoter methylation [160]. TNFα upregulates c-myc transcription via β-catenin during the progression from BE to EAC. On top of that, EAC can be influenced by upregulation of WNT2, loss of WIF1, and promoter hypermethylation of sFRP1 and APC genes, while HGF and TNFβ can induce nuclear accumulation of β-catenin in esophageal cells [158,162].
- **Implication in EAC-TME:** (i) Warburg effect; (ii) nutritional supply promoting cell survival; (iii) recruitment of immunosuppressive cells; (iv) reduced cytotoxic T-cell activity and NK tumor cytolytic effect; (v) increased hypoxia and angiogenesis; (vi) increased glycolysis [163,164,165].
- **Druggable target:** NCT00006245 trial evaluated the combination of Flavopiridol (cyclin-dependent kinase (CDK) inhibitor) in c-Myc positive ECs [166], while NCT02884453 [167] evaluated the combination of HER2 and c-Myc targeted agents for those cases that present HER2 and c-Myc overexpression. In addition, AXL receptor tyrosine kinase inhibitors are promising agents for suppressing tumors that present c-Myc overexpression [168].

### 4.2. Pathological Signaling Pathways in EAC

#### 4.2.1. Insulin-like Growth Factor 1 Receptor (IGF1R) Pathway, with IGF1R Being a Tyrosine Kinase, Is Robustly Activated in EAC

- **Frequency:** It has been demonstrated that there is an increased pIRS1 staining (IGF1R activation marker) in 43.2% of BE patients and 70% of EAC patients, a phenomenon that implies the significant effect of IGF1R in BE progression towards dysplasia and EAC [158].
- **Physiological function:** Typically, it has a key role in cell growth, proliferation, and differentiation, as well as survival and several cell metabolic functions.
- **Pathological function in EAC:** This pathway is activated when insulin receptor substrate 1 (IRIS1/2) is phosphorylated, with the phosphorylation of IGF1R that is implicated in RAS/RAF/MEK/ERK and PI3K/AKT/mTOR downstream signal transduction [169].
- **Druggable target:** Monoclonal antibodies (mAbs) against IGF1R, such as dalotuzumab (MK-0646), cixutumumab (IMC-A12), ganitumab (AMG-479), and figitumumab (CP-751, 871) have been evaluated in EAC [170]. More particularly, the phase 2 ECOG-ACRIN E2208 trial (NCT01142388), in which cixutumumab (IMC-A12) combined with Paclitaxel, did not show improvement of OS and PFS versus paclitaxel in patients with metastatic EC or GEJ adenocarcinoma [171]. Figitumumab (CP-751, 871) was also evaluated in a phase 2 trial, NCT0062610, in advanced solid tumors, including EC, with the trial being terminated by the sponsor [172]. In contrast, a combination of Ganitumab and chemotherapy was evaluated in GI cancer (phase 2, NCT00819169), without noteworthy results [173]. Another drug modality for modulating this signaling pathway is the IGF1R inhibitor, which is a tyrosine kinase inhibitor (TKI). Phase 1 NCT01016860 trial, in which linsitinib (OSI-906) was combined with irinotecan in GI tumors, including EC, was terminated due to halted OSI-906 development [174].

#### 4.2.2. c-MET

- **Frequency:** It is significantly upregulated in BE and EAC, exhibiting c-MET positivity in 100% of dysplastic BE and EAC patients.
- **Physiological role:** a tyrosine kinase receptor activated by hepatocyte growth factor (HGF).
- **Abnormal function in EAC:** The overexpression of c-MET has been closely related to unfavorable prognosis and shorter OS [175].
- **Druggable target:** Several clinical trials evaluated c-MET inhibition in advanced gastric or EAC, such as phase 1/2 NCT02344810 and phase 2 NCT (NCT02016534) trial, in which oral AMG 337 (c-MET inhibitor) utilization was assessed [176]. Likewise, the combination of tivantinib (c-MET Inhibitor) with FOLFOX has also been evaluated in NCT01611857 for patients with advanced solid tumors, including EAC, which presented a favorable effect on advanced GEJ [177]. This combination has shown favorable results for PFS in patients with advanced EAC and GEJ compared to the first-line FOLFOX treatment strategy [177]. Interestingly, the phase 1 NCT03205501 trial evaluated EMI-137, a fluorescent c-MET agent for imaging, for its utilization in BE, dysplasia, and EAC endoscopic identification, serving as a potential screening and monitoring tool in EAC and esophageal dysplasia [178].

#### 4.2.3. Notch Signaling

- **Frequency:** It has been demonstrated that NICD expression is high in approximately 72% of total EAC cases, promoting uncontrolled cell proliferation, drug resistance to chemotherapeutic agents, as well as angiogenesis, acting as an oncogene.
- **Abnormal function in EAC:** It is increasingly recognized as a critical oncogenic driver contributing to various hallmarks of cancer progression. This signaling pathway plays a pivotal role in the maintenance of CSC populations and the differentiation of EAC cells. Altered activation of Notch signaling has been associated with enhanced tumorigenicity and chemoresistance.
- **Druggable target:** Suppression of Notch signaling has demonstrated a great potential in EAC cell sensitization to chemotherapy [155,156,157]. Focusing on the pivotal function of this signaling pathway, Notch1–Notch4 transmembrane receptors on the surface of cells interact with Jagged (JAG1 and 2) and Delta-like (DLL1, 3, and 4) ligands, a phenomenon that triggers Notch receptor cleavage, which subsequently leads to Notch intracellular domain (NICD) release. Afterward, NICD enters the nucleus and induces the activation of HES and HEY genes, regulating cell proliferation and differentiation, as well as homeostasis [158]. Meanwhile, it prevents uncontrolled proliferation and abnormal differentiation via KLF4-dependent mechanisms, thereby suppressing BE progression towards EAC [179,180,181,182,183].

#### 4.2.4. Hh Signaling Pathway

- **Frequency:** Its dysregulation is also identified in esophageal intestinal metaplasia. This signaling pathway is activated not only in a large number of EAC cases, but also in BE that progress to EAC.
- **Abnormal function in EAC:** This activation occurs when the Patched (PTCH) receptor interacts with Hh ligands, leading to the removal of Smoothened (SMO) protein inhibition. This, in turn, further activates and upregulates Gli transcription factors (GLI1, GLI2, GLI3) in the nucleus. The activation of this signaling is closely associated with cancer cell growth, disease progression, and drug resistance [184,185]. Meanwhile, it has been demonstrated that there is an enhanced staining for Hh-related proteins (96% of EAC tissues), while 90% of EAC patients have an altered Gli1/2 expression. Moreover, it has been demonstrated that the Hh pathway is normally inactive in normal esophageal epithelium. At the same time, acid reflux induces intestinal metaplasia, resulting in BE development. Hh signaling may potentially promote BE by increasing the expression of BMP4, FOXA2, and SOX9 transcription factors; however, it is associated with notably decreased expression in physiological epithelium, compared to GERD-related BE cases [184,185,186]. The overexpression of these transcription factors promotes metaplasia, leading to BE progression to EAC, as well as increasing tumor growth, angiogenesis, and chemoresistance. Meanwhile, cancer cell survival is amplified via the interaction between Hh and mTOR pathways, which occurs under the condition of chronic inflammation of the esophageal epithelium (acid reflux) [184,185,186].
- **Druggable target:** Hh signaling has been evaluated as a druggable target, in case of its dysregulation, in several clinical trials such as the Itraconazole trial (phase 2, NCT05563766), in which itraconazole (antifungal) was evaluated as an Hh pathway suppressor, demonstrating favorable results regarding the prevention of BE progression towards EAC [187]. Another preclinical study evaluated the combination of Hh pathway inhibitor (GDC-0449) with mTOR inhibitor (RAD-001), which demonstrated a notable reduction in the esophageal cancer cell volume [188].

In Table 3, we summarize all the genetic aberrations that serve as druggable targets in clinical trials for EC, including EAC.

### 4.3. Epigenetic Aberrations in EAC

Epigenetic aberrations are also demonstrated in EAC, which induce alterations in gene expression without changing the DNA sequence. These modifications include DNA methylation, chromatin remodeling, and non-coding RNAs (e.g., short non-coding RNAs (microRNAs), long non-coding RNAs (lncRNAs)), which influence gene expression and act as tumor promoters or suppressors, as well as histone modifications.

- **Histone modifications**, such as ubiquitination, phosphorylation, acetylation, deacetylation, and methylation, can lead to an altered chromatin accessibility, resulting in gene silencing or transcription [189]. Histone hypoacetylation has been demonstrated in EAC, leading to the silencing of tumor suppressor genes, with histone deacetylases (HDACs) overexpression resulting in dysregulated chromatin accessibility, altered tumor cell proliferation, and survival.
- **DNA hypermethylation of tumor suppressor genes** leads to their silencing and genomic instability, a phenomenon that promotes oncogenesis and dysfunctional DNA repair. Some of the examples of DNA methylation in EAC include the hypermethylation of the CDKN2A promoter that leads to p16INK4a silencing, which is observed in 45–68% of BE cases and in 70–80% of EAC, promoting the uncontrolled cell cycle [190,191]. RUNX3 hypermethylation is also reported, which leads to the silencing of this gene that acts as a tumor suppressor, facilitating the progression from BE to EAC [161]. Similarly, RASSF1A, APC, and E-cadherin promoter methylations have also been reported, which lead to cell cycle dysregulation and apoptosis suppression for the former, β-catenin accumulation for the second, while invasion and metastatic dissemination for the latter [161,192]. In addition, the methylations of RUNX3 and APC have been closely correlated with an increased risk of disease progression BE to EAC, whereas that of CDKN2A is related to dysplasia, having a potential role as a biomarker in early stages of dysplasia that may lead to progression from BE to EAC [161].
- **MiRNAs and lncRNAs dysregulations** have been reported in EAC, such as the upregulation of lncRNA H19, miR-21, miR-196a, and miR-192, which have an oncogenic role leading to increased EAC cell growth, proliferation, and invasion, drug resistance to chemotherapy (miR-21), as well as disease progression from BE to EAC and worrisome prognosis (miR-192, miR-21). Among them, miR-21 is closely associated with several signaling pathways such as PI3K/AKT, Ras-Raf-MEK-ERK, and EGFR, while it is also implicated in apoptosis. Meanwhile, the downregulation of other miRNAs that have a key role as tumor suppressors has also been demonstrated in EAC, such as in the case of miR-200, leading to EMT and distant dissemination [193,194]. It has also been shown that miR-526B targets, e.g., hedgehog-interacting protein (HHIP), deleted in azoospermia (DAZ)1–4, as well as aristaless-related homeobox (ARX), are downregulated during the BE progress towards EAC. This aberration could serve as a druggable target via miRNA modulation [195]. Meanwhile, ARID1A deletions or mutations are attributed to the defective chromatin, leading to an abnormal DNA repair mechanism and silencing of this gene, which usually has a tumor suppressive role [141,142]. In Table 4 we present some examples of the epigenetic aberrations in EAC.

## 5. Future Perspectives

TME modulation in EAC constitutes a promising strategy for enhancing anti-tumor immunity and overcoming drug resistance. The results of several clinical trials have proven that this therapeutic approach is quite promising. Some novel approaches include the utilization of exosome-mediated drug delivery or vaccines, with the latter utilizing exosomes that are embedded with cargoes aiming at inducing an anti-tumor immune response in the TME. Tumor-derived exosomes/large oncosomes, which carry several pro-tumor cargoes such as MMPs, growth factors, tumor-promoting miRNAs or lncRNAs that modify ECM and suppress anti-tumor immunity, could serve as therapeutic targets. Several circulating exosomal miRNAs are closely associated with tumor progression in BE towards EAC, with a panel of them being a potential diagnostic tool for differentiation between healthy esophageal tissue and BE versus EAC, including ratios of miRNAs such as miR-30a-5p/miR-324-5p, miR-25-3p/miR-320a, RNU6-1/miR-16-5p, miR-17-5p/miR-194-5p, as well as let-7e-5p/miR-15b-5p. It has been demonstrated that overexpression of miR-194, miR-199 family, miR-223, miR-21, and miR-196, as well as miR-25, is closely related to BE progression. In contrast, miR-203, -205, -378, etc., have a tumor-suppressive role, are downregulated [196]. It has been demonstrated that loading exosomes with miR-1-3p suppresses the MAPK/ERK signaling pathway via targeting E2F5 in preclinical EC models [197]. Meanwhile, miR-17-5p (downregulated) could also be targeted as it is correlated with resistance to chemoradiotherapy in EAC models [198]. Likewise, acidic bile salts (ABS) EV-EGFRs associated with the M2 phenotype in EAC models could also be utilized as druggable targets [199]. More particularly, it has been demonstrated that ABS-related exosomal EGFR regulates M2 polarization, inducing and facilitating EAC proliferation [200]. EVs can also serve as a drug delivery system (engineered nanocarriers), exhibiting high biocompatibility. However, further preclinical and clinical studies are required [200].

Furthermore, taking into consideration the beneficial role of ICIs in addition to chemotherapy as first-line EAC treatment (e.g., Nivolumab, pembrolizumab, atezolizumab), emerging combinations between ICs (dual), or the addition of TIGIT inhibitors to ICIs (potential synergy) and chemotherapy, constitute novel approaches that are currently evaluated to minimize drug resistance and improve OS rates. Meanwhile, perioperative or neoadjuvant immunotherapy is under study, with promising results [201].

The utilization of oncolytic viruses as cancer vaccines is also in the spotlight, aiming at the enhancement of anti-neoplastic immune responses of DC and cytotoxic T cells in TME. More particularly, a Japanese phase 1 trial (UMIN000007541) evaluated OBP-301/telomelysin. Telomerase-specific oncolytic adenovirus was administered via endoscopic intratumoral injection with concurrent radiotherapy in non-resectable EC or in patients ineligible for chemotherapy. Interestingly, this strategy presented an objective response rate of 91.7% [202]. It has also been histologically demonstrated that T cells were significantly increased in cases of partial response. Meanwhile, complete response has been reported in 60% of stage 2 and 3, and 83.3% of stage 1 [202]. NRG-GI007 (Phase I, ongoing, NCT04391049) is another trial that also evaluates telomelysin, combined with chemoradiation (weekly dosing of paclitaxel or carboplatin plus radiotherapy) in locally advanced, non-resectable EC/GEJ patients [203].

H101 (oncorine), which is an E1B-55 kDa gene-deleted adenovirus with selective replication within cancer cells, has been evaluated in phase 3 (NCT00879385) in combination with chemotherapeutic (cisplatin-based plus 5-FU) in ESCC cases, as well as squamous cell cancer of the head and neck. This study presented a notable increase in ORR, compared to chemotherapy alone, although with no data regarding its administration in EAC [204]. Meanwhile, the NCT07061704 phase 1/2 trial that is currently ongoing evaluates the combination of an oncolytic virus with ICI and chemotherapy in ESCC patients with non-resectable, locally advanced tumors, although with no clinical data regarding EAC [205].

Another preclinical study evaluated the efficacy of IFN-expressing Oncolytic Adenovirus (Cox2-promoter, Ad5/3-pCDX2) infection and destruction of EAC cells, which express CDX2. CDX2 is a transcription factor implicated in intestinal metaplasia (IM) in EAC. The aforementioned modality presented a notable synergistic effect with 5-FU concurrent administration not only in vivo but also in vitro [206]. In addition, G47Δ herpes simplex virus (3d-generation) preclinical study in mice EC models (orthotopic), which was intratumorally injected, suppressed the tumor growth. However, further studies are required to evaluate its possible synergistic effect with chemo- or immunotherapy [207]. NCT06910657, a phase 1 trial that is currently recruiting, evaluates the efficacy of IDOV-Immune, an oncolytic vaccinia virus, in patients with advanced solid tumors, including EC [208].

Although no chimeric antigen receptor T (CAR-T) cell therapy has been currently approved for EAC therapy, there have been several preclinical and clinical trials performed. HER2-targeted CAR-T cells have also been evaluated in xenograft ESCC mouse models. The combination CAdVEC (Chimeric Adenoviral Vector Encoding Chemokine), which is an oncolytic adenovirus, and HER2-specific chimeric antigen receptor (CAR) T cells (genetically modified), which express a receptor against the HER2 antigen on tumor cells (HER2-positive), has been evaluated in NCT03740256, a phase 1 trial for patients with HER2+ advanced solid tumors, including EC [209]. This oncolytic virus electively infects tumor cells and not only induces but also enhances the anti-tumor effect of these CAR T cells. Likewise, phase 1 NCT03618381 is the first human study that evaluates the efficacy of autologous CAR T cells against EGFR in patients with advanced solid tumors, including EC [210]. A Phase 1 clinical trial (NCT03706326) evaluates the effectiveness of PD-1 gene knockout (PD-1 KO) MUC1-targeted CAR-T in patients with advanced EC, but mainly ESCC, with an OS of 24 months for those who completed multiple infusions. Likewise, MUC1-targeted, CD276-targeted CAR-T cells have also been evaluated in preclinical animal models and humans, respectively. MUC1-directed CAR-T cells administration demonstrated favorable results regarding the duration and the intensity of anti-tumor activity in EC models in vitro and in vivo [211]. CD276-specific CAR-T cells also demonstrate favorable anti-tumor effects and improved survival in mouse models, as CD276 is overexpressed in EC tissue. Last but not least, microbiome modulation constitutes another promising strategy (fecal transplantation, probiotics, synbiotics, as well as antibiotics) for EAC management and TME modulation, as the gut microbiome is significantly implicated in EAC progression and drug resistance [212]. More particularly, NCT05412628 observational study compares the microbiota in EC and oropharyngeal cancers, to identify any microbial pattern that leads to EAC development, radiation, and chemotherapy resistance. Meanwhile, it evaluates whether probiotics could potentially improve the esophageal or oral microbiota when dysbiosis is present [213]. However, there are not many interventional studies regarding microbiome modulation in EC, including EAC. The combination of fecal microbiota transplantation (FMT) with immunotherapy in non-resectable EAC is evaluated in the Biorich2 (NCT03706326) trial, which is actively recruiting [214]. Nevertheless, further studies are necessary to assess the efficacy of FMT, synbiotics, and probiotics administration aiming at microbiome modulation in EAC patients. In Table 5. We demonstrate some of the future therapeutic approaches in EC treatment.

## 6. Conclusions

EC remains a highly aggressive malignancy with a poor prognosis, drug resistance, and reduced OS. Despite the multimodal therapeutic approaches that have notably improved disease outcomes, including tumor resection, chemotherapy, immunotherapy, targeted therapies, and radiotherapy, OS remains suboptimal. TME plays a key role in disease progression and metastasis, with its modulation being the focus of several studies. Several emerging therapeutic approaches for TME modulation, such as microbiome-targeted therapies, oncolytic vaccines, and CAR-T cell therapy, EV-based miRNAs, could be promising for overcoming drug resistance and enhancing anti-tumor immune response. Integration of several therapeutic modalities and their evaluation in preclinical and clinical trials will open new horizons for novel therapeutic approaches that could improve disease outcomes.

## Figures and Tables

**Table 1:** TME components and their implication in TME [7,8,9,10,11,12,13,14,15,16,17,18,19,20,21,22,23,24,25,26,27,28,29,30,31,32,33,34,35,36,37,38,39,40,41,42,43,44,45,46,47,48,49,50].

Type of TME Content	Subcomponents	Role in TME
Non-cellular ECM	Collagen types I, II, IV (type I most abundant)	Structural support, bridging gaps between cancer/stromal cells; supports cell proliferation, adhesion, invasion, and migration; storage of growth factors (FGF, VEGF, TGF-β, PDGF); dense ECM prevents drug delivery → resistance.
	Elastin	Provides elasticity.
	Glycoproteins (fibronectin, vitronectin, laminin, integrins)	Promote tumor cell adhesion and migration.
	E-cadherin	Downregulation: weak adhesion, invasion, migration, and dissemination.
	Tenascin-C Osteopontin	Facilitates adhesion, migration, and metastatic dissemination, Facilitates cancer cell survival.
	Glycosaminoglycans, Proteoglycans	It is implicated in signaling pathways and the storage of growth factors.
	MMP-2, MMP-9, from stroma cells & adipocytes	ECM remodeling promotes proliferation, angiogenesis, and migration; Overexpressed: poor prognosis.
Cellular	CAFs	Secrete VEGF, TGF-β → immunosuppressive niche, invasiveness (MMP-3 degradation of E-cadherin), drug resistance. Also secretes TGF-β → suppresses distant dissemination.
	MDSCs	Promote growth & progression via angiogenesis (MMPs, VEGF, cytokines), EMT (IL-6, TGF-β, S100A8/A9) Suppressing T cells promotes Tregs (tumor immune escape) Secrete TNF-α, IL-10, IL-1β, enhancing inflammation. Promote invasion/migration/metastasis. Interact with CSCs, influencing their survival, resistance, and self-renewal.
	Cancer stem cells	Migratory/invasive, proliferative (signaling/genetic changes), survival via releasing anti-apoptotic proteins, immune escape, drug resistance via releasing efflux pumps, resistant proteins
	B-cells	Produce antibodies against tumor neoantigens, aiming for cancer elimination.
	Bregs	Tumor-promoting; suppresses innate immune cells.
	CD8^+^ cytotoxic T cells	Identify tumor neoantigens (TCR + MHC-I), induce lysis, Overexpression: favorable prognosis.
	CD4^+^ helper T cells (Th1)	Anti-tumor effects; secrete TNF-α, IFN-γ → stimulate CD8^+^ and M1 macrophages recruitment
	CD4^+^ helper T cells (Tregs)	Express CTLA-4, PD-1, FOXP3; secrete IL-2, IL-10, IL-35, TGF-β. Suppress CD8^+^ and NK cells, interact with ECs, CAFs, stromal cells → progression & angiogenesis.
	CD4^+^ helper T cells (Th2)	Promote M2 macrophages (pro-tumor phenotype), enhance immunosuppression.
	CD4^+^ helper T cells (Th17)	Dual role: promote angiogenesis/inflammation/progression, or act as suppressors.
	DCs	Present tumor neoantigens to T cells, recruit immune cells, secrete IFN-α/β (anti-tumor effect). Immature DCs exhibit impaired APC function, tumor immune escape, and induce Tregs.
	Neutrophil-N1 phenotype	Anti-tumor phenotype (early stage): release ROS, TNF-α, ICAM-1 → immune cell recruitment, tumor elimination
	Neutrophil-N2 phenotype	Pro-tumor phenotype; release VEGF (angiogenesis), MMP-9 (invasion, metastasis), T cell suppression
	TAMs	M1 phenotype: anti-tumor: acts as APC, interacts with and stimulates CD8^+^ T cells M2 phenotype: pro-tumor, secretion of cytokines/angiogenic mediators. Overexpressed M2: poor prognosis.
	Natural killer cells	Direct lysis of tumor cells via perforin, granzymes, recognize low MHC-I and secrete IFN-γ for immune cell recruitment Dysregulated: cancer progression.
	ECs	Mediate angiogenesis (VEGF), EMT modulation (TGF-β, IL-6, VEGF, PDGF). Secrete VEGF, PDGF, FGF → leaky vessels (extravasation, metastasis). Express PD-L1 (immune suppression), Express ICAM-1 bound to LFA-1on T cells (impaired immune response), Express VCAM-1 bound to VLA-4 on T cells (immune migration, exploited mechanism for metastasis)
Soluble mediators		
EVs	Large oncosomes (1000–10,000 nm)	Oncogenic cargo from tumor cells induces TME modification and tumor progression.
	Oncosomes (100–400 nm) Exosomes (50–150 nm), microvesicles (150–1000 nm)	Carry growth factors (TGF-β1, VEGF), non-coding RNAs, oncogenes; promote progression, migration, ECM remodeling, immune modulation (Tregs, cytotoxic T cells), neoangiogenesis, metastasis, drug resistance.
Growth factors	VEGF	Induces new “leaky” vessel formation (neoangiogenesis).
	TGF-β	Promotes immunosuppression.
	PDGF	Induces ECM remodeling.
	EGF, FGF	Enhance cancer cell proliferation, survival, invasion, and stromal modification.
Metabolic mediators/tumor metabolites	MMPs	Promote ECM degradation, tumor invasion, and metastasis.
	IDO	Induces immunosuppression.
		Suppresses cytotoxic T cell function.
Chemokines	CCL2, CCL5, CCL9, CCL10, CCL12	Upregulated chemokines recruit tumor-promoting immune cells (TAMs, Tregs, MDSCs).
	CXCL9, CXCL10	Downregulated → reduced recruitment of cytotoxic NK and T cells → tumor immune evasion.
Cytokines	TGF-β, IL-10, IL-6 (from Tregs)	Tumor-promoting cytokines suppress anti-tumor immunity.
	IFN-γ, TNF-α (from CD8^+^ and NK cells)	Tumor-suppressing cytokines enhance anti-tumor immunity.
Hypoxia		Activated energy conservation pathways for tumor cells’ survival Hypoxia-sensing systems trigger biological responses that influence cell survival, resulting in treatment-resistant tumor subpopulations or cell death.

Extracellular Matrix (ECM), Cancer-Associated Fibroblasts (CAFs), Myeloid-Derived Suppressor Cells (MDSCs), Cancer Stem Cells (CSCs), B-Regulatory Cells (Bregs), T-Regulatory Cells (Tregs), Dendritic Cells (DCs), Tumor-Infiltrating Lymphocytes (TILs), Tumor-Associated Macrophages (TAMs), Natural Killer Cells (NK), Endothelial Cells (ECs), Matrix Metalloproteinases (MMPs), Fibroblast Growth Factor (FGF), Vascular Endothelial Growth Factor (VEGF), Transforming Growth Factor-β (TGF-β), Platelet-Derived Growth Factor (PDGF), Epidermal Growth Factor (EGF), Epithelial–Mesenchymal Transition (EMT), Antigen-Presenting Cells (APCs), Cytotoxic T-Lymphocyte Antigen-4 (CTLA-4), Programmed Cell Death Protein-1 (PD-1), Programmed Death-Ligand 1 (PD-L1), Forkhead Box P3 (FOXP3), Interferon-γ (IFN-γ), Interleukin (IL), Tumor Necrosis Factor-α (TNF-α), Reactive Oxygen Species (ROS), Intercellular Adhesion Molecule-1 (ICAM-1), Vascular Cell Adhesion Molecule-1 (VCAM-1), Lymphocyte Function–Associated Antigen-1 (LFA-1), Very Late Antigen-4 (VLA-4), Extracellular Vesicles (EVs), Indoleamine 2,3-Dioxygenase (IDO), Chemokine (C-C motif) Ligand (CCL), Chemokine (C-X-C motif) Ligand (CXCL).

**Table 2:** TME modulation and therapeutic strategies, including preclinical (**a**) and (**b**) clinical studies [19,32,51,52,53,54,55,56,57,58,59,60,61,62,63,64,65,66,67,68,69,70,71,72,73,74,75,76,77,78,79,80].

**(a) Preclinical studies**				
**Strategy/Target**	**Drug(s)**	**Trial/Study**	**Population/Design**	**Key Results/Status**
CAF-targeting (PDE5 inhibition)	PDE5 inhibitors	Preclinical	EAC fibroblast/CAF models; PDE5i + SOC chemotherapy	Blocked CAF transdifferentiation, overcame chemoresistance
CAF-targeting (IL-6 blockade)	Tocilizumab	Preclinical	EAC TME; tocilizumab + chemo/radiation	Reduced growth, reversed immunosuppression, resensitized tumors
CAF-targeting (NOX4 inhibition)	NOX4 inhibitors	Preclinical	CAF-rich EAC models	Reduced CAF activation and immunoresistance
MDSC-targeting (CXCR2 antagonists)	CXCR2 inhibitors	Preclinical	EAC TME	Suppressed EMT, angiogenesis, metastasis
TAM-targeting (CSF1R inhibition)	CSF1R inhibitors	Preclinical	EAC TME	TAM depletion/reprogramming
IDO1 inhibition	IDO1 inhibitors	Preclinical	EAC TME	Reduced T-cell suppression; reversed immune evasion
Integrin blockade (ITGA6)	None (gene knockout)	Preclinical	ECs in vitro	Knockout of ITGA6 reduced proliferation and invasion
**(b) Clinical studies**				
**Strategy/Target**	**Drug(s)**	**Trial (Phase/ID)**	**Population/Design**	**Key Results/Status**
PD-1 + chemo	Pembrolizumab	KEYNOTE-590 (Phase 3, NCT03189719)	Advanced EAC & ESCC; pembrolizumab + chemo vs. chemo	Improved PFS & OS; supports 1st-line pembrolizumab
PD-1 + CRT	Pembrolizumab	KEYNOTE-975 (Phase 3, NCT04210115)	EAC; pembrolizumab + CRT vs. placebo + CRT	Ongoing; no results yet
PD-1 perioperative	Pembrolizumab	KEYNOTE-585 (Phase 3, NCT03221426)	Resectable GEJ/GC	Did not meet the OS endpoint
PD-L1 + FLOT	Durvalumab	MATTERHORN (Phase 3, NCT04592913)	Resectable GEJ/GC	Improved OS & pCR
PD-L1 + FLOT	Atezolizumab	DANTE (Phase 2/3, NCT03421288)	Resectable GEJ/GC	Benefit esp. in PD-L1+ & MSI-high
PD-L1 + TIGIT + chemo	Atezolizumab + Tiragolumab	MORPHEUS-EC (Phase 1b/2, NCT03281369)	Advanced EC	Improved survival & tumor response
PD-L1 + TIGIT + chemo	Atezolizumab + Tiragolumab	SKYSCRAPER-08 (Phase 3, NCT04540211)	Advanced ESCC	Increased OS
PD-1 + chemo	Nivolumab	CheckMate-649 (Phase 3)	GEJ/EAC/GC, HER2–	Improved OS
PD-1 ± LAG-3	Nivolumab ± Relatlimab	NCT03044613 (Phase 2)	Resectable stage II–III EC/GEJ	Arm A safe; Arm B toxic; better response in LAG-3/PD-L1 high
PD-L1 + CRT	Atezolizumab	PERFECT (Phase 2, NCT03044613)	E/GEJ; atezolizumab + CRT	No significant difference; baseline IFNγ predicted benefit
PD-1/TIGIT + chemo	Domvanalimab + Zimberelimab	STAR-221 (Phase 3, NCT05568095)	Advanced GEJ/EAC	Ongoing; no results yet
PD-1/TIGIT + chemo	Domvanalimab + Zimberelimab	EDGE-Gastric (Phase 2, NCT05329766)	GEJ/EAC/GC	Higher ORR with the combination
VEGF + chemoRT	Bevacizumab	NCT00570531 (Phase 2)	Pre-op EAC	Terminated early
VEGF (ziv-aflibercept + chemo)	Ziv-Aflibercept	ZAMEGA (Phase 2, NCT01747551)	Refractory GC/GEJ	No benefit
VEGF + chemo	Bevacizumab	Phase 2	Untreated metastatic GEJ/GC	Improved OS & PFS
VEGFR2 + chemo	Ramucirumab	NCT01246960 (Phase 2)	Advanced EAC/GEJ/GC	Higher OS (exposure–response)
VEGF + chemo	Bevacizumab	AVAGAST (Phase 3)	Locally advanced/metastatic GEJ/GC	OS benefit: predictive biomarkers (VEGF-A, NRP-1)
VEGFR2 + paclitaxel	Ramucirumab	RAINBOW (Phase 3, NCT01170663)	Advanced GEJ/GC	Improved OS (2nd line)
VEGFR2 monotherapy	Ramucirumab	REGARD (Phase 3, NCT00917384)	Advanced GEJ/GC	OS benefit
VEGFR2 + PD-1	Ramucirumab + Pembrolizumab	JVDF (Phase 1a/1b, NCT02443324)	GEJ; 2nd line	Superior in PD-L1+
VEGFR2 + PD-1	Ramucirumab + Pembrolizumab	NCT04632459 (Phase 2)	Metastatic GC/GEJ	Ongoing
VEGF + PD-1 + chemo	Bevacizumab + Anti-PD-1	NCT06415851 (Phase 2)	Advanced EC	Recruiting
VEGF + PD-1 + HDAC inhibitor	Bevacizumab + Anti-PD-1 + Tucidinostat	NCT05163483 (Phase 2)	Advanced MSS/MMR EC	Recruiting

Cancer-Associated Fibroblasts (CAFs), Tumor Microenvironment (TME), Esophageal Adenocarcinoma (EAC), Esophageal Squamous Cell Carcinoma (ESCC), Gastroesophageal Junction (GEJ), Gastric Cancer (GC), Immune Checkpoint Inhibitor(s) (ICI), Programmed Death-1 (PD-1), Programmed Death-Ligand 1 (PD-L1), Cytotoxic T-Lymphocyte–Associated Protein 4 (CTLA-4), T-cell Immunoreceptor with Ig and ITIM Domains (TIGIT), Lymphocyte-Activation Gene 3 (LAG-3), Tumor-Associated Macrophages (TAMs), Myeloid-Derived Suppressor Cells (MDSCs), Dendritic Cells (DCs), Cancer Stem Cells (CSCs), Epithelial-to-Mesenchymal Transition (EMT), Extracellular Matrix (ECM), Objective Response Rate (ORR), Complete Response (CR), Duration of Response (DOR), Progression-Free Survival (PFS), Overall Survival (OS), Disease-Free Survival (DFS), Treatment-Related Adverse Events (TRAEs), Microsatellite Instability (MSI), Mismatch Repair (MMR), Chemoradiotherapy (CRT), Standard of Care (SOC), Fluorouracil (5-FU), Folinic Acid/Leucovorin (LV), Oxaliplatin (O), Docetaxel (T), FLOT regimen (5-FU, Leucovorin, Oxaliplatin, Docetaxel), FOLFOX regimen (5-FU, Leucovorin, Oxaliplatin), CAPOX regimen (Capecitabine, Oxaliplatin).

**Table 3:** Genetic aberrations as druggable targets in clinical trials for EAC [42,85,86,87,88,89,90,91,92,93,94,95,96,97,98,99,100,101,102,103,104,105,106,107,108,109,110,111,112,113,114,115,116,117,118,119,120,121,122,123,124,125,126,127,128,129,130,131,132,133,134,135,136,137,138,139,140,141,142,143,144,145,146,147,148,149,150,151,152,153,154,155,156,157,158,159,160,161,162,163,164,165,166,167,168,169,170,171,172,173,174,175,176,177,178,179,180,181,182,183,184,185,186,187,188].

Gene/Pathway	Location	Function/Role	Aberration in EAC	Prevalence/Notes	Trials
TP53	17p13.1	Tumor suppressor: genomic stability, cell cycle, apoptosis, oxidative stress regulation	Loss-of-function mutation; LOH; altered p53 expression	~70% of EAC; wild-type in ~30%	Reactivation with PRIMA-1, APR-246; combined with cisplatin + 5-FU (NCT02999893, NCT04383938)
HER2/ERBB2	17q12	Receptor tyrosine kinase; cell growth, differentiation, survival (MAPK/PI3K-AKT)	Amplification; overexpression	15–30% in EAC; 28% in HGD	Trastuzumab, Pertuzumab, T-DM1, DS-8201a (DESTINY-Gastric01 NCT03329690; DESTINY-Gastric05 NCT06731478; JACOB trial NCT01358877)
VEGFA	6p21.1	Angiogenesis; tumor proliferation	Overexpression; mutations	30–60% in metastasized EAC	Anti-VEGF agents (clinical trials as mentioned)
CDKN2A (p16INK4a/p14ARF)	9p21	Tumor suppressor; cell cycle regulation via CDK4/6 and p53	Deletion, mutation, promoter hypermethylation	20–30% in EAC; 15–60% hypermethylation	CDK4/6 inhibitors (palbociclib, abemaciclib, ribociclib); epigenetic reactivation (azacitidine, NCT01386346)
KRAS	12p12.1	Proto-oncogene; RAS-RAF-MEK-ERK signaling	Mutation; amplification	Amplification in ~17% of EAC	Pan-KRAS inhibitors (LY4066434, NCT06607185; BI 3706674, NCT06056024)
SMAD4	18q21.2	Tumor suppressor; TGF-β/SMAD signaling, ECM, differentiation, apoptosis	Mutation, deletion, LOH	5–10% of EAC	BMP2/4 inhibition in SMAD4-negative EAC (preclinical, VHH antibodies)
ARID1A	1p36.11	Tumor suppressor; chromatin remodeling, DNA repair	Mutation: loss of expression	9–15% of EAC; 4.9–16% in dysplastic BE/HGD	ICIs (tremelimumab, durvalumab, NCT05023655); statins + chemo + nivolumab
PIK3CA	—	PI3K/AKT/mTOR signaling	Mutation, amplification	5–15%	PI3K inhibitors (BKM120 NCT01501604, Inavolisib NCT04551521, PF-07248144 NCT04606446)
MYC	8q24	Transcription factor: cell cycle, metabolism, proliferation	Amplification, overexpression	45.3%	CDK inhibitors (Flavopiridol NCT00006245); combination with HER2-targeted therapies (NCT02884453); AXL inhibitors
IGF1R	—	Tyrosine kinase: growth, proliferation, metabolism	Upregulation; increased pIRS1 staining	43.2% in BE; 70% in EAC	Monoclonal antibodies: dalotuzumab, cixutumumab, ganitumab, figitumumab (NCT01142388, NCT0062610, NCT00819169, NCT01016860)
c-MET/HGF receptor	—	Tyrosine kinase; growth, survival	Overexpression, activated by HGF	100% in dysplastic BE and EAC	AMG 337 (NCT02344810, NCT02016534); Tivantinib + FOLFOX (NCT01611857); EMI-137 imaging (NCT03205501)
Notch signaling/NICD	—	CSC maintenance, differentiation	Overactivation; NICD upregulation	~72% of EAC	Suppression enhances chemosensitivity (preclinical)
Hedgehog (Hh)/PTCH-SMO-GLI	—	Cell proliferation, disease progression	Overactivation; GLI1/2 altered	96% Hh proteins; 90% altered Gli1/2	Itraconazole (NCT05563766), GDC-0449 + RAD-001 (preclinical)

TP53—Tumor protein p53; HER2/ERBB2—Human epidermal growth factor receptor 2/Erb-b2 receptor tyrosine kinase 2; VEGFA—Vascular endothelial growth factor A; CDKN2A—Cyclin-dependent kinase inhibitor 2A; KRAS—Kirsten rat sarcoma viral oncogene homolog; SMAD4—Mothers against decapentaplegic homolog 4; ARID1A—AT-rich interactive domain-containing protein 1A; PIK3CA—Phosphatidylinositol-4,5-bisphosphate 3-kinase catalytic subunit alpha; MYC—Myelocytomatosis oncogene/c-Myc; IGF1R—Insulin-like growth factor 1 receptor; c-MET/HGFR—Mesenchymal–epithelial transition factor/Hepatocyte growth factor receptor; NICD/Notch—Notch intracellular domain/Notch signaling pathway; Hh/PTCH-SMO-GLI—Hedgehog/Patched-Smoothened-GLI signaling pathway.

**Table 4:** Epigenetic aberrations in EAC [141,142,161,189,190,191,192,193,194,195].

Gene/Pathway	Chromosome/Location	Function/Role	Aberration in EAC	Prevalence/Notes	Therapeutic Implications/Trials
CDKN2A (p16INK4a)	9p21	Tumor suppressor; cell cycle regulation	Promoter hypermethylation: silencing	45–68% in BE, 70–80% in EAC	Biomarker for dysplasia; early diagnostic/prognostic value
RUNX3	1p36	Tumor suppressor; regulates differentiation & apoptosis	Promoter hypermethylation: silencing	Associated with BE EAC progression	Potential biomarker; epigenetic therapy target
RASSF1A	3p21	Tumor suppressor; apoptosis regulation	Promoter methylation: silencing	Reported in EAC	Candidate for demethylating therapy
APC	5q21	Tumor suppressor; Wnt/β-catenin regulation	Promoter methylation: inactivation	Reported in EAC	β-catenin accumulation; Wnt-targeted therapies
E-cadherin (CDH1)	16q22	Cell adhesion; invasion suppression	Promoter methylation: loss of expression	Reported in EAC	Promotes invasion/metastasis; epigenetic modulation
HDACs		Chromatin remodeling; transcription regulation	Overexpression: histone hypoacetylation	Leads to altered proliferation & survival	HDAC inhibitors (clinical trials ongoing)
lncRNA H19	—	Oncogenic lncRNA promotes proliferation.	Upregulation	Reported in EAC	Associated with progression & drug resistance
miR-21	17q23.2	Oncogenic miRNA; apoptosis & proliferation	Upregulation	Linked to BE EAC progression, poor prognosis	Impacts PI3K/AKT, Ras-Raf-MEK-ERK, EGFR; drug resistance
miR-192	11q13.1	Oncogenic miRNA; proliferation & invasion	Upregulation	Associated with BE EAC progression, poor prognosis	Potential biomarker; inhibition strategies
miR-196a	12q13.13	Oncogenic miRNA; tumor progression	Upregulation	Reported in EAC	Therapeutic inhibition strategies
miR-200 family	1p36, 12p13, others	Tumor suppressor miRNAs; EMT regulation	Downregulation	Reported in EAC	Leads to EMT/metastasis; restoration therapy
miR-526B	19q13.42	Tumor suppressor miRNA; targets HHIP, DAZ1–4, ARX	Downregulation	Observed in BE → EAC progression	Potential druggable target via miRNA modulation
ARID1A	1p36	Chromatin remodeling; DNA repair; tumor suppressor	Deletions/mutations: defective chromatin remodeling	Reported in EAC	Synthetic lethality approaches (e.g., EZH2 inhibitors)

CDKN2A—Cyclin-dependent kinase inhibitor 2A; RUNX3—Runt-related transcription factor 3; RASSF1A—Ras association domain family member 1; APC—Adenomatous polyposis coli; CDH1—Cadherin 1 (E-cadherin); HDACs—Histone deacetylases; lncRNA H19—Long non-coding RNA H19; miR−21—MicroRNA-21; miR-192—MicroRNA-192; miR-196a—MicroRNA-196a; miR-200—MicroRNA-200 family; miR-526B—MicroRNA-526B; ARID1A—AT-rich interaction domain 1A.

**Table 5:** Future therapeutic approaches in EC treatment [196,197,198,199,200,201,202,203,204,205,206,207,208,209,210,211,212,213,214].

Target/Pathway		Function/Role	Aberration in EAC	Notes/Prevalence	Therapeutic Implications/Trials
Telomerase (TERT)	5p15.33	Maintains telomere length, supports tumor growth	Targeted in non-resectable EC	Overexpressed in most EAC tumors	OBP-301/telomelysin oncolytic adenovirus; Phase I trials (UMIN000007541, NRG-GI007, NCT04391049)
H101 (Oncorine)	E1B-55kDa-deleted adenovirus	Selective tumor replication	Not tested in EAC; ESCC and HNSCC tested	Phase III trial in ESCC	Combined with cisplatin + 5-FU (NCT00879385)
IFN-expressing Oncolytic Adenovirus (Ad5/3-pCDX2)	Cox2-promoter adenovirus	Tumor-selective lysis, cytokine expression	Targets CDX2+ EAC cells	Preclinical	Synergistic with 5-FU in vitro and in vivo; potential for combination therapy
G47Δ HSV-1	Herpes simplex virus, 3rd generation	Oncolytic viral therapy	Suppresses tumor growth	Orthotopic EC mouse models	Potential combination with chemo- or immunotherapy
IDOV-Immune (Vaccinia virus)	Oncolytic virus	Anti-tumor immune activation	Advanced solid tumors, including EC	Phase I trial (NCT06910657)	Safety and preliminary efficacy evaluation
HER2		Oncogenic receptor tyrosine kinase	Overexpressed in HER2+ EC	Preclinical xenograft models	HER2-specific CAR-T cells; CAdVEC + CAR-T (NCT03740256)
EGFR		RTK promotes proliferation	Overexpressed in some EC	Phase I trials	EGFR-targeted CAR-T cells (NCT03618381)
MUC1		Tumor-associated antigen	Overexpressed in EC; PD-1 KO enhances CAR-T efficacy	Preclinical + Phase I (NCT03706326)	CAR-T therapy; improved OS in multiple infusions
CD276 (B7-H3)		Immune checkpoint ligand	Overexpressed in EC	Preclinical	CAR-T therapy improved survival in mouse models
Exosomal miRNAs	Circulating EVs	Regulate tumor growth, ECM, and immunity	Oncogenic miRNAs upregulated (miR-194, miR-21, miR-196, miR-25); tumor-suppressive miRNAs downregulated (miR-203, miR-205, miR-378)	Panels used for diagnosis and monitoring progression	Therapeutic targeting via exosome delivery; modulation of TME signaling
miR-1-3p	Exosomes	Tumor suppressor, inhibits MAPK/ERK via E2F5	Downregulated in EAC	Preclinical	Exosome-mediated delivery to suppress proliferation
miR-17-5p	Exosomes	Oncogenic, chemoradiotherapy resistance	Upregulated in resistant EAC models	Preclinical	Targeted therapy to overcome resistance
EV-EGFR (Exosomal)	Exosomes from acidic bile salts	Induces M2 macrophage polarization, promotes proliferation	Upregulated in EAC	Preclinical	Targetable to modulate immune TME
Immune Checkpoints (PD-1, PD-L1, TIGIT)	TME/immune cells	Inhibit T cell function	Dysregulated	Contributes to immune evasion and drug resistance	Dual ICIs, TIGIT inhibitors, and neoadjuvant immunotherapy trials
Oncolytic viruses as vaccines	Viral vectors (OBP-301, telomelysin)	Activate DCs and cytotoxic T cells	Enhances anti-tumor immunity	Phase I trials	Intratumoral injection combined with chemoradiation; objective response rates up to 91.7%
Microbiome modulation	Gut/oral microbiota	TME and immune modulation	Dysbiosis contributes to EAC progression and therapy resistance	Observational and early interventional trials (NCT05412628, Biorich2 NCT03706326)	Fecal microbiota transplantation, probiotics, synbiotics, and antibiotics; potential synergy with immunotherapy

PD-L1—Programmed Death-Ligand 1; PD-1—Programmed Death-1; TME—Tumor Microenvironment; TERT—Telomerase Reverse Transcriptase; ESCC—Esophageal Squamous Cell Carcinoma; EAC—Esophageal Adenocarcinoma; HNSCC—Head and Neck Squamous Cell Carcinoma; CAR-T—Chimeric Antigen Receptor T cells; RTK—Receptor Tyrosine Kinase; EV—Extracellular Vesicle; ICIs—Immune Checkpoint Inhibitors; FMT—Fecal Microbiota Transplantation; ABS—Acidic Bile Salts; DC—Dendritic Cells.

## Data Availability

No new data were created or analyzed in this study. Data sharing is not applicable to this article.

## Abbreviations

5-FU	5-Fluorouracil
AKT	Protein kinase B
APC	Adenomatous polyposis coli
ARID1A	AT-rich interaction domain-containing protein 1A
BE	Barrett’s esophagus
CAFs	Cancer-Associated Fibroblasts
CAPOX regimen	Capecitabine, Oxaliplatin
CAR-T	Chimeric Antigen Receptor T cells
CDK	Cyclin-dependent kinase
CDKN2A	Cyclin-dependent kinase inhibitor 2A
CR	Complete Response
CRT	Chemoradiotherapy
CSC/CSCs	Cancer Stem Cell(s)
CTLA-4	Cytotoxic T-Lymphocyte
CTL	Cytotoxic T Lymphocyte
DCs	Dendritic Cells
DFS	Disease-Free Survival
DOR	Duration of Response
EAC	Esophageal Adenocarcinoma
EC	Esophageal Cancer
ECM	Extracellular Matrix
EGFR	Epidermal Growth Factor Receptor
EMT	Epithelial-to-Mesenchymal Transition
ERBB2/HER2	Human Epidermal Growth Factor Receptor 2
ESCC	Esophageal Squamous Cell Carcinoma
EV	Extracellular Vesicles
FLOT regimen	5-FU, Leucovorin, Oxaliplatin, Docetaxel
FMT	Fecal Microbiota Transplantation
FOLFOX regimen	5-FU, Leucovorin, Oxaliplatin
GC	Gastric Cancer
GEJ	Gastroesophageal Junction
HES	Hairy/Enhancer of Split
HEY	Hairy/Enhancer of Split-related
HGF	Hepatocyte Growth Factor
HNSCC	Head and Neck Squamous Cell Carcinoma
HSV-1	Herpes Simplex Virus Type 1
ICI/ICIs	Immune Checkpoint Inhibitor(s)
IHC	Immunohistochemistry
IFN	Interferon
IGF1R	Insulin-like Growth Factor 1 Receptor
IRS1/2	Insulin Receptor Substrate 1/2
IT	Intratumoral
KO	Knockout
KRAS	Kirsten rat sarcoma viral oncogene homolog
LAG-3	Lymphocyte-Activation Gene 3
LOH	Loss of Heterozygosity
LV	Leucovorin (Folinic Acid)
MAPK/ERK	Mitogen-Activated Protein Kinase/Extracellular Signal-Regulated Kinase
miRNA	MicroRNA
MDSCs	Myeloid-Derived Suppressor Cells
mFOLFOX6	Modified FOLFOX6 (fluorouracil, leucovorin, oxaliplatin)
miR	microRNA (specific subtypes listed in text)
MMR	Mismatch Repair
mTOR	Mechanistic Target of Rapamycin
MSI	Microsatellite Instability
MSS	Microsatellite-Stable
MUC1	Mucin 1
MYC/c-Myc	Myelocytomatosis oncogene protein
NICD	Notch Intracellular Domain
Obs	Observational
ORR	Objective Response Rate
OS	Overall Survival
OV	Oncolytic Virus
PD-1	Programmed Death-1
PD-L1	Programmed Death-Ligand 1
PFS	Progression-Free Survival
PI3K	Phosphoinositide 3-Kinase
PIK3CA	Phosphatidylinositol-4,5-bisphosphate 3-kinase catalytic subunit alpha
RASSF1A	Ras Association Domain Family Member 1
Rb	Retinoblastoma protein
RNU6-1	Small nuclear RNA U6-1
RT	Radiotherapy
RTK	Receptor Tyrosine Kinase
sFRP1	Secreted Frizzled-Related Protein 1
SOC	Standard of Care
SWI/SNF	Switch/Sucrose Non-Fermentable chromatin remodeling complex
TAMs	Tumor-Associated Macrophages
TGF-β	Transforming Growth Factor Beta
TGFBR2	Transforming Growth Factor Beta Receptor 2
TIGIT	T-cell Immunoreceptor with Ig and ITIM Domains
TNFα	Tumor Necrosis Factor Alpha
TME	Tumor Microenvironment
TP53	Tumor protein p53
TRAEs	Treatment-Related Adverse Events
VEGFA	Vascular Endothelial Growth Factor A
WIF1	Wnt Inhibitory Factor 1
